# Constitutive Metabolite Profiling of European and Asian *Fraxinus* with Varying Susceptibility to Ash Dieback

**DOI:** 10.1007/s10886-025-01678-z

**Published:** 2026-01-15

**Authors:** Beatrice Tolio, Patrick Sherwood, Diana Marčiulynienė, Christoph Crocoll, Michelle Cleary, Mateusz Liziniewicz

**Affiliations:** 1 Skogforsk - The Forest Research Institute, Ekebo 2250, Svalöv, 268 90 Sweden; 2 Southern Swedish Forest Research Centre, Swedish University of Agricultural Sciences, Sundsvägen 3, Alnarp, 234 56 Sweden; 3 Institute of Forestry, Lithuanian Research Centre for Agriculture and Forestry, Liepų str. 1, Girionys, Kaunas district, LT-53101 Lithuania; 4 Department of Plant and Environmental Sciences, University of Copenhagen, Thorvaldsensvej 40, Frederiksberg C, 1871 Denmark

**Keywords:** European ash, *Hymenoscyphus fraxineus*, Defence chemistry, Metabolomics, Coumarins, Flavonoids

## Abstract

*Hymenoscyphus fraxineus* is an invasive pathogen native to East Asia, responsible for the widespread mortality of European ash (*Fraxinus excelsior*) throughout Europe. Asian ash species, which co-evolved with *H. fraxineus*, are considered more tolerant than European ash. However, within European ash populations, a small proportion of genotypes show low susceptibility to the pathogen. This study sought to characterize the underlying defence mechanisms to *H. fraxineus* by performing untargeted constitutive metabolomics profiling of phloem and leaf tissue of from thirteen *F. excelsior* genotypes (nine tolerant and four susceptible) and five genotypes representing three Asian ash species (*F. mandshurica*, *F. platypoda*, and *F. chinensis*). Here we report 57 and 36 compounds associated with lower or higher disease susceptibility, from phloem and leaf tissue, respectively. Flavonoids and coumarins were the main classes of detected compounds. In particular, quercitrin and fraxetin exhibited greater variation among the groups. In phloem tissue, quercitrin and fraxetin were more abundant in tolerant than in susceptible European ash and, lowest in Asian ash species. In leaves, however, quercitrin was highest in Asian ash, followed by tolerant and then susceptible European ash. Other flavonoids, coumarins, and iridoid glycosides also showed variation among groups, with stronger differences in phloem than in leaf tissue. Overall, this study advances our understanding of metabolite composition in *Fraxinus* species with different co-evolutionary histories and susceptibility to *H. fraxineus* and demonstrates the potential of untargeted metabolomics for investigating defence-related mechanisms in plant-pathogen interactions.

**Supplementary Information:**

The online version contains supplementary material available at 10.1007/s10886-025-01678-z.

## Introduction

Forest ecosystems worldwide are threatened by invasions of non-native phytophagous insects and phytopathogens (PIPs), that can cause dramatic ecological damage and economic losses (Bonello et al. 2020; Showalter et al. 2018). Such invasions are rapidly increasing due to globalization, in particular the trade of plants for planting or movement of wood products, which facilitates the long-distance movement of species into regions outside their natural distribution range (Prospero and Cleary 2017). Additionally, climate change is likely to contribute to the expansion of both native and exotic pests and pathogens by creating more favourable environmental conditions and/or by altering their behaviour and impact (Ramsfield et al. 2016). The lack of co-evolutionary history between native host and introduced pests and pathogen is widely suggested as the main reason why non-native PIPs can cause such devastating damage to their host (Bonello et al. 2020; Stenlid and Oliva 2016). Within these interactions, native hosts can be highly susceptible, although some individuals display resistance - preventing infection - or tolerance - enduring infection with limited impact (Budde et al. 2016). Characterizing how non-native PIPs interact with host species in introduced ranges will help in making decisions and strategies for PIPs management, including mitigating impacts with tree resistance breeding programs.

In Europe, European ash (*Fraxinus excelsior* L.) is currently under threat from an epidemic disease, known as ash dieback (ADB). The disease was initially observed in Lithuania and Poland in the early 1990 s (Juodvalkis and Vasiliauskas 2002; Kowalski and Holdenrieder, 2009), however, there was apparently a time lag of several years between the introduction of the disease and the first reports of symptoms and mortality (Carroll and Boa 2024). Currently, the disease is widespread throughout most of European ash’s natural range and has dramatically reduced the host population size (Pautasso et al. 2013; Timmermann et al. 2011). The causal agent of the disease is the ascomycete fungus *Hymenoscyphus fraxineus* (Kowalski) Baral, Queloz, Hoya (Baral et al. 2014; Kowalski 2006; Kowalski and Holdenrieder 2009). The pathogen probably arrived in Europe on nursery stock and/or seeds of Asian ash (e.g. *F. mandshurica*) that had been introduced to Europe for amenity planting during the previous century (Cleary et al. 2016; Drenkhan et al. 2014). Population genetic analyses of *H. fraxineus* isolates from asymptomatic *F. mandshurica* have revealed a larger allelic diversity in Asian than in European population, suggesting that the pathogen is native to East Asia and likely experienced a bottleneck effect during its introduction to Europe (Cleary et al. 2016; Gross et al. 2014a, b). In European ash, *H. fraxineus* is known to infect both leaves and phloem tissue. The infections start on leaves by the pathogen ascospores. Following leaf infection, the mycelium spread through the petiole into shoots, twigs, branches and sometimes main stems, causing necrotic cankers (Haňáčková et al. 2017). However, in its natural Asian hosts, the fungus primary colonizes leaves, with limited progression into woody tissue (Nielsen et al. 2017).

Currently, there is no effective method to eradicate this established pathogen. However, there is evidence that a small proportion of ash genotypes show tolerance to the pathogen. These genotypes appear to be healthy trees, showing lower disease symptoms even in environments with high disease pressure and significant tree mortality. Studies conducted throughout Europe show that low susceptibility to the disease is under strong genetic control, inheritable, polygenic, and stable over time (Enderle et al. 2015; Kjær et al. 2012; Liziniewicz et al. 2022; McKinney et al. 2011; Munõz et al. 2016; Pliura et al. 2011; Pliūra et al. 2014; Stener 2018; Stocks et al. 2019). The genetic foundation of tolerance to ash dieback suggests that selective breeding may be an effective strategy to mitigate disease´s impacts. However, conventional breeding practices are time-consuming, as they involve long life spans and generations of tree species and are laborious, as a large number of genotypes require screening and testing in field trials. Genetic marker-assisted selection may facilitate early screening of trees with favourable traits and accelerate the breeding process (Chaudhary et al. 2020). Recent works utilized associative transcriptomics and whole genome sequencing to identify molecular markers associated with different levels of susceptibility of European ash populations. These studies successfully predicted and identified individuals with low susceptibility to the disease (Chaudhary et al. 2020; Harper et al. 2016).

Similar approaches using chemical makers are being explored. Metabolite profiling is an ideal tool to distinguish between groups of trees that vary in disease susceptibility and to identify metabolites correlated to disease resistance and tolerance (Conrad et al. 2023). This is because plant resistance against pests and pathogens relies on a wide variety of host defence mechanisms, ranging from physical defences to chemical defences, the latter based on plant secondary metabolites. Metabolite-based defence strategies involve both constitutive and inducible mechanisms. Constitutive metabolites are produced regardless of the presence of external threats, while inducible defences are triggered in response to external stimuli or stressors (Witzell and Martín 2008). Previous studies have been able to distinguish between tolerant and susceptible *F. excelsior* genotypes and have identified putative metabolites associated with either phenotype. Cleary et al. (2014) reported qualitative and quantitative differences in the constitutive and induced metabolite composition of *F. excelsior* genotypes seedlings originating from individuals previously phenotyped as being more or less susceptible to *H. fraxineus*. In the untargeted metabolite profiling of Sambles et al. (2017) and in the targeted approach of Sidda et al. (2020), putative constitutive iridoid glycosides were found to differ between resistant and susceptible genotypes from ash leaf extracts. More recently, Nemesio-Gorriz et al. (2020) identified fraxetin and esculetin as two coumarins present at constitutive levels and strongly associated with reduced susceptibility to ash dieback. Additionally, the role of both compounds was further supported using in-vitro assays that inhibited the growth of the pathogen (Nemesio-Gorriz et al. 2020). These studies suggest that the identification and use of chemical biomarkers holds vast potential as a screening tool for plant material in breeding programs.

All the referenced studies have focused on understanding constitutive host-defence mechanisms in European ash that are associated with low susceptibility to ash dieback. However, the role of constitutive chemical defences in ash species that co-evolved with the pathogen remains largely unknown. Research on these phytochemicals in Asian ash species could significantly enhance our understanding of host-pathogen interactions and factors influencing disease susceptibility in the European ash population, the outcome of which could expedite the discovery of biomarkers associated with broad-based tolerance and facilitate disease control.

This study sought to (i) characterize the metabolite composition of *Fraxinus* species that differ in their co-evolutionary history with *H. fraxineus*, reflecting inter-specific variation in tolerance, and (ii) examine the metabolite composition associated with intra-specific variation in tolerance within *F. excelsior* genotypes that exhibit different susceptibility to ash dieback. We performed untargeted liquid chromatography-mass spectrometry (LC-MS) analyses of constitutive secondary metabolites of leaf and phloem tissue from European ash genotypes differing in their degree of susceptibility to ash dieback and from three different Asian ash species. We hypothesize that: (i) the levels of constitutive metabolites in Asian ash species differ from European ash; (ii) these compounds will be at higher amount in European ash genotypes with reduced susceptibility to *H. fraxineus*.

## Methods and Materials

### Study Site and Sampling

The material for this study was selected from a clonal field trial established in 2016 in Snogeholm, Sjöbo, Sweden (55°32’57.4"N 13°42’29.4"E). The trial consisted of 65 replicated ash genotypes: 56 tolerant *F. excelsior* trees selected from around Sweden, four known susceptible *F. excelsior* genotypes, and five genotypes representing three *Fraxinus* species of Asian origin. The Asian ash include: *F. mandshurica* native to China, Japan, Korea, Eastern Russia; *F. platypoda* native to China and Japan and, *F. chinensis* native to northern China, Korea, Japan, south-east Russia (Wallander 2013). Visually healthy leaf and shoot samples (including bark and phloem) were collected in June 2020 from 13 *F. excelsior* genotypes varying in disease severity to *H. fraxineus* (9 tolerant-ET and 4 susceptible-ES) based on periodic assessments, and from all five resistant Asian ash genotypes (AT). From one to five ramets (replicates) per genotype were collected depending on the amount of available tissue (Table S1). A total of 92 samples were collected in the field, 46 leaves and 46 phloem from the current year shoots. Collected samples were placed in plastic bags and kept on dry ice then stored at −80 °C until further processing.

### Sample Preparation

Samples and tools were kept frozen in liquid nitrogen during all processing steps to minimize degradation or tissue oxidation. Bark including phloem was peeled from the twigs with a sterile scalpel. Bark and leaf tissue were ground separately in liquid nitrogen with porcelain mortars and pestles. For each sample, 100 mg of ground tissue was lyophilized for 24 h. Metabolite extraction was performed by incubating the tissue statically for 24 h at 4 °C with 500 µL of high-performance liquid chromatography grade MeOH containing 5 µM schaftoside (Phytolab item no. 8332, Vestenbergsgreuth, Germany) as an internal standard. Samples were then centrifuged (4000 x *g*, 2 min), and the supernatant was transferred to a new 2 mL microcentrifuge tube and stored at −20 °C. The pellet was re-extracted as described above and the new supernatant was combined with previous extraction to get approximately 1000 µL of extract total per sample.

### Untargeted Metabolomics by LC-MS/Q-TOF

Samples were 5-fold diluted with 75% MeOH in water and subjected to analysis by ultra performance liquid chromatography coupled to tandem mass spectrometry (UPLC-MS/MS) at the Department of Plant and Environmental Sciences, University of Copenhagen. UPLC-MS/MS analysis was performed on a Dionex UltiMate 3000 Quaternary Rapid Separation UHPLC^+^ focused system (Thermo Fisher Scientific, Germering, Germany). Separation was achieved on a Kinetex 1.7 μm XB-C18 column (100 × 2.1 mm, 1.7 μm, 100 Å, Phenomenex). For eluting 0.05% (v/v) formic acid in H_2_O and acetonitrile [supplied with 0.05% (v/v) formic acid] were employed as mobile phases A and B, respectively. Gradient conditions were as follows: 0.0− 1.0 min 3% B; 1.0− 40.0 min 3 − 30% B; 40.0− 50.0 min 30 − 60% B, 50.0− 53.0 min 60 − 100% B, 53.0− 56.0 min 100% B, 56.0− 56.5 min 100 − 3% B, and 56.5− 60.0 min 3% B. The flow rate of the mobile phase was 300 µL/min. The column temperature was maintained at 30 °C. The UHPLC was coupled to a Compact micrOTOF-Q mass spectrometer (Bruker, Bremen, Germany) equipped with an electrospray ion source (ESI) operated in negative ion mode. The ion spray voltage was maintained at −3900 V in negative ion mode. Dry temperature was set to 250 °C, and the dry gas flow was set to 8 L/min. Nitrogen was used as the dry gas, nebulizing gas, and collision gas. The nebulizing gas was set to 2.5 bar and collision energy to 15 eV. MS spectra were acquired in an *m/z* range from 50 to 1000 *m/z* at a sampling rate of 3 Hz. MS/MS spectra were acquired in a range from 200 to 900 *m/z*. Na-formate clusters were used for mass calibration. MS raw data were processed with mzMine software version 2.53 (Pluskal et al. 2010) for peak detection, peak deconvolution, peak alignment and gap filling. The resulting peak-list were tentatively identified by matching values found in the KEGG COMPOUND Database (Kanehisa 2016).

### Data Analysis

Venn diagrams were constructed to visualize the number of metabolites unique and shared among tolerant European ash (ET), susceptible European ash (ES) genotypes and Asian ash species (AT) using the ggvenn package (Yan and Yan 2021). The diagrams were generated with all detected features prior to data preprocessing. Before multivariate analysis, data were filtered using the “modified 80% rule” to reduce the effects of missing value by removing features that had more than 20% missing data in any phenotype group (Yang et al. 2015). The remaining missing values were replaced using a KNN (K-nearest neighbour) missing value imputation, followed by cantering and Pareto scaling (Yang et al. 2015). Differences in metabolic profiles among tolerant European ash (ET), susceptible European ash (ES) genotypes and Asian ash species (AT) were analysed by partial square discriminant analysis (PLS-DA), using mixOmics package (Rohart et al. 2017). PLS-DA models were tuned with a 5-fold, 100 repeat cross-validated balanced error rate values. Since PLS-DA has a high propensity to overfitting, all PLS-DA models were validated with permutation tests using RVAideMoire package (Hervé 2019; Hervé et al. 2018). PLS-DA was applied to identify the most influential compounds for discriminating between the different groups. Compound importance was determined based on variable importance in the projection (VIP) scores (Lê Cao and Welham 2021). Metabolites with VIP > 1 were considered as potential features that contribute to the separation among tolerant European ash (ET), susceptible European ash (ES) genotypes and Asian ash species (AT). Shapiro-Wilk test was applied on the residuals of the selected metabolites. To test for differences in the abundance of each metabolite, data with normally distributed residuals were subjected to one-way analyses of variance (ANOVA) with Tukey post hoc test, while data with non-normally distributed residuals were subjected to Kruskal-Wallis with Dunn post hoc test. P-values from ANOVA and Kruskal-Wallis test were subjected to Benjamini-Hochberg correction to control for the false discovery rate (Benjamini and Hochberg 1995). Significance was considered at a threshold of *p* < 0.05. Data and statistical analysis were performed using RStudio version 2024.12.0.467 (RStudio Team 2025).

## Results

### Phloem Tissue

**Fig. 1:**
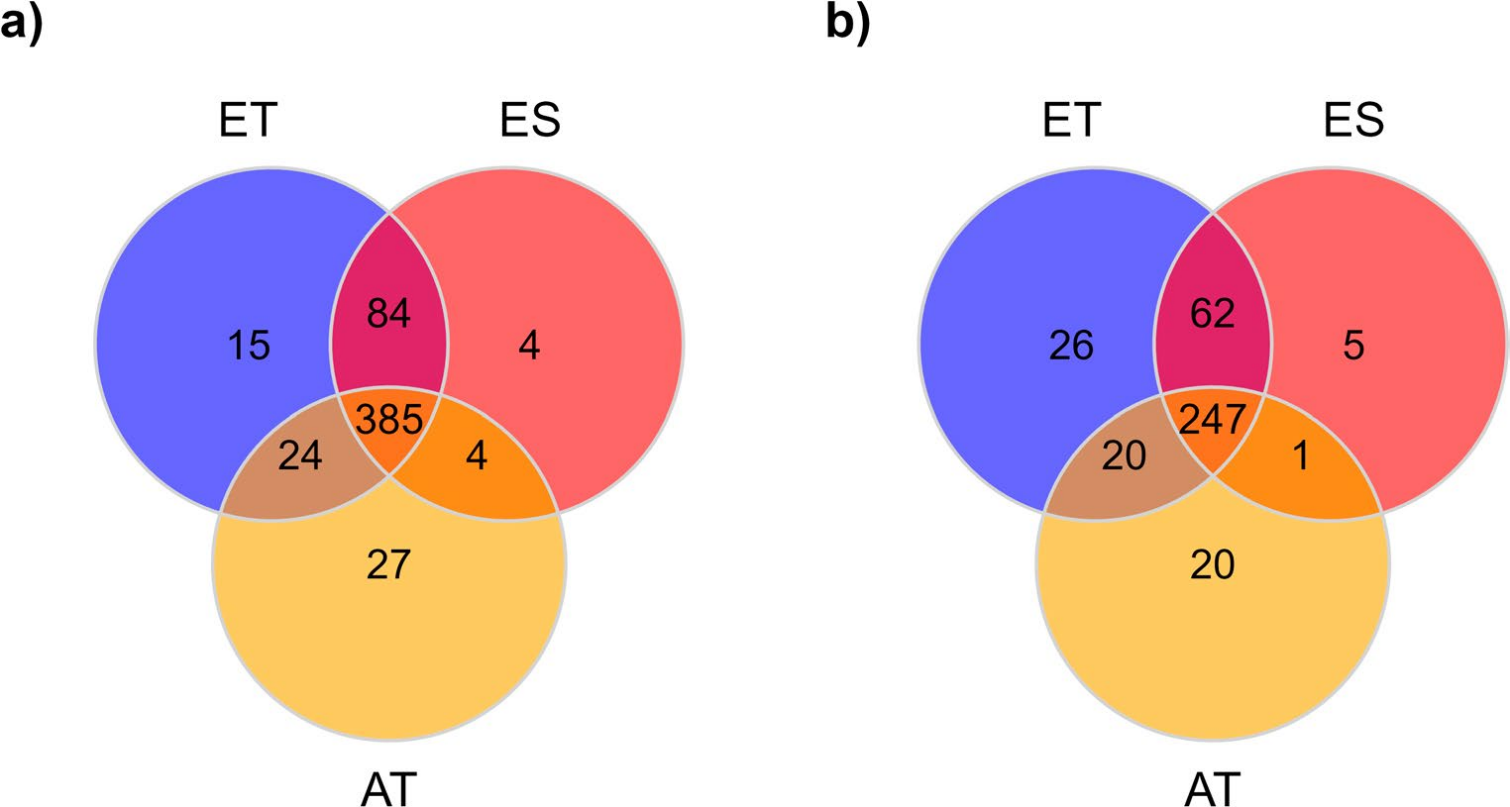
Venn diagrams representing the number of chemical features detected in Asian ash species (AT), susceptible European ash (ES), and tolerant European ash (ET) from (**a**) phloem tissue and (**b**) leaf tissue

**Fig. 2:**
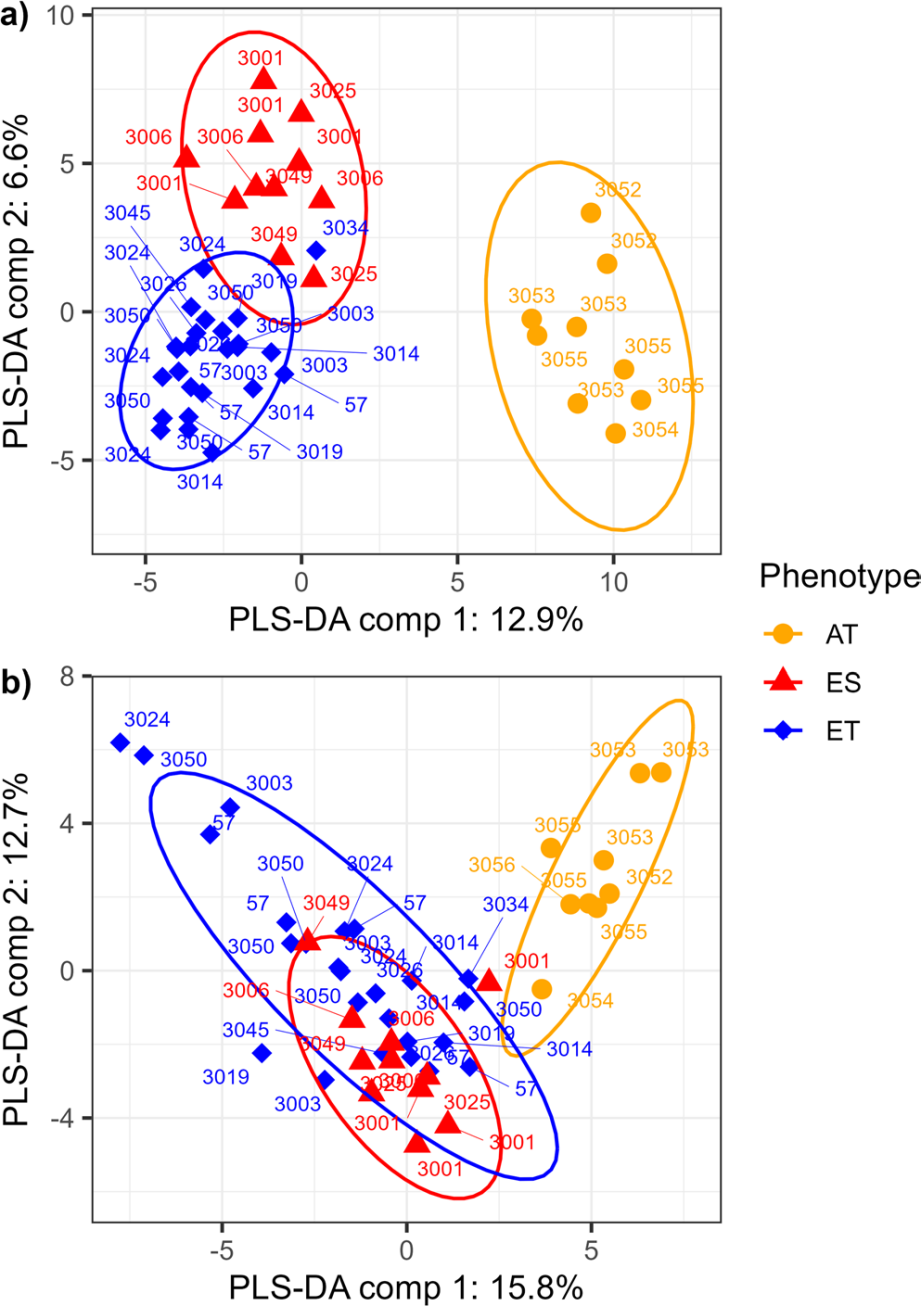
Confidence ellipse plots (95% confidence level) of partial square discriminant analysis (PLS-DA) discriminating metabolites profiles of Asian ash species (AT), susceptible European ash (ES), and tolerant European ash (ET) from (**a**) phloem tissue and (**b**) leaf tissue. Label numbers indicate the corresponding genotype ID

**Fig. 3:**
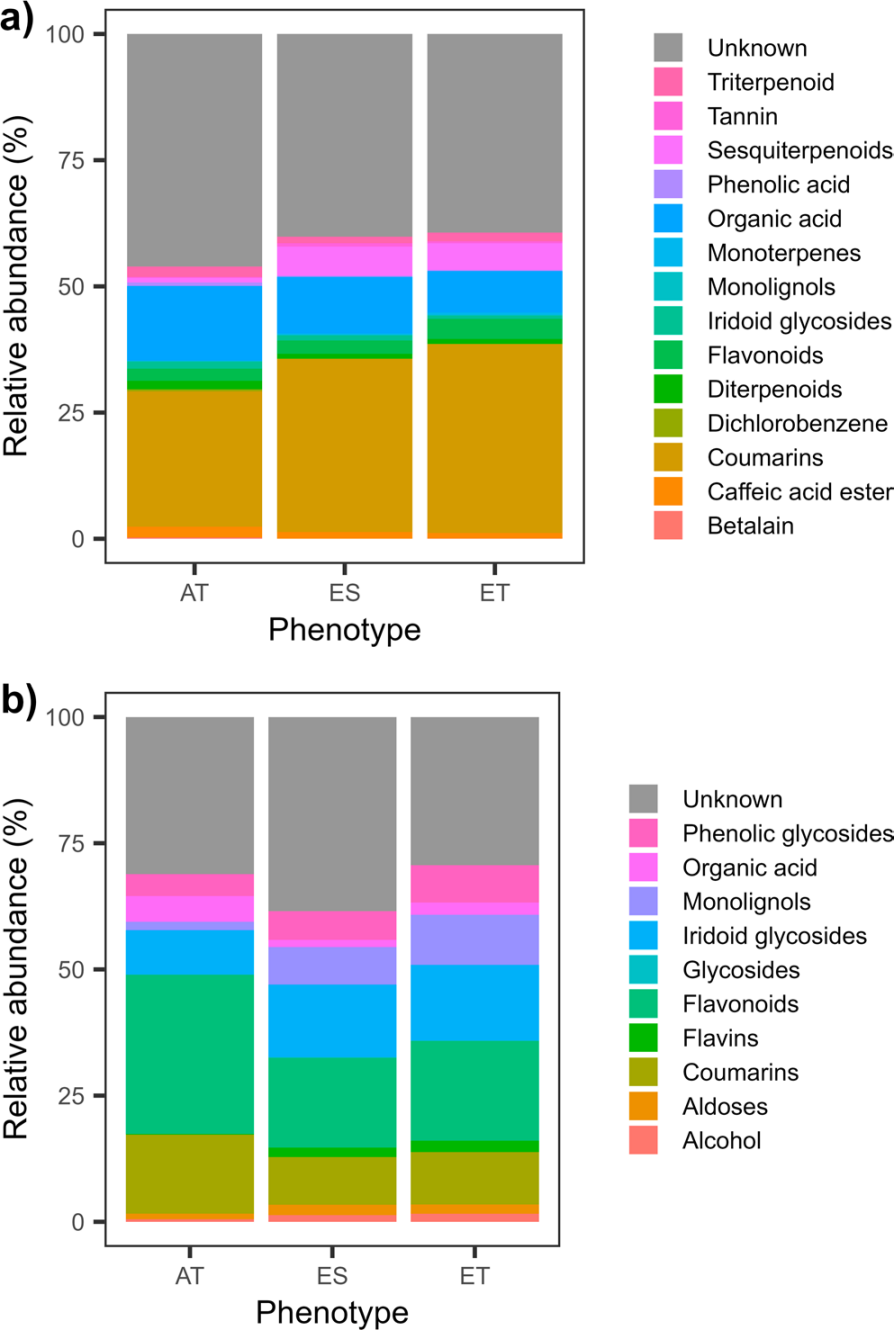
Relative abundance of the biochemical classes of the most informative chemical features identified with LC-MS/Q-TOF and PLS-DA analysis from (**a**) phloem tissue and (**b**) leaf tissue

A total of 543 features were detected with untargeted LC-MS analysis in the phloem samples (Fig. 1a). A full list of these features is presented in Table S2. Of these, approximately 71% of the features were shared among susceptible European ash (ES), tolerant European ash (ET) and Asian ash species (AT). PLS-DA enabled a clear discrimination among susceptible European ash (ES), tolerant European ash (ET) and Asian ash species (AT) based on their metabolite profiles (Fig. 2a). PLS-DA revealed that the first component accounted for 12.9% and the second component accounted for 6.6% of the total variance. The most important features were identified for the first PLS-DA component, which explained the most variance and contributed to the best separation between groups. Based on VIP scores, fifty-seven features were identified as being important in explaining the differences among the three groups (Table 1). All fifty-seven features were present in the three different phenotype groups, regardless of the missing value imputation. Compounds detected from ash phloem tissue included flavonoids, coumarins, iridoid glycosides, monoterpenes, diterpenoid, triterpenoids, and phenolic acids (Fig. 3a).

**Table 1:** Mean (± standard error of the mean) ion counts (x10^2^) of the most informative chemical features in phloem tissue identified by identified by LC-MS/Q-TOF and PLS-DA analysis. Metabolites are listed in decreasing order of variable importance in the projection (VIP) scores

Feature number	[M-H]^–^(m/z)	Rt (min)	Putative ID	Biochemical class	VIP	AT	ES	ET	Statistical tests*
C167	354.0585	12.09	Unknown	-	2.48	6.0 (± 0.4) b	19.7 (± 1.4) a	21.3 (± 0.7) a	K
C405	619.1657	22.98	Unknown	-	2.46	40.9 (± 5.9) a	7.2 (± 0.3) b	6.5 (± 0.3) b	K
C127	389.1086	10.69	Monotropein	Iridoid glycosides	2.39	59.5 (± 5.5) a	28.6 (± 3.7) b	19.2 (± 1.7) b	A
C420	531.1131	23.89	Biochanin A 7-O-(6-O-malonyl-beta-D-glucoside)	Flavonoids	2.17	22.7 (± 2.6) a	9.4 (± 1.8) b	8.0 (± 0.7) b	K
C181	820.1175	12.95	Unknown	-	2.14	10.5 (± 0.8) b	21.2 (± 0.6) a	19.5 (± 0.6) a	A
C244	415.1602	16.00	Unknown	-	2.10	36.4 (± 3.0) a	19.4 (± 1.6) b	21.1 (± 0.6) b	K
C166	207.0305	12.09	Fraxetin	Coumarins	2.07	87.5 (± 2.5) c	116.7 (± 4.6) b	143.5 (± 5.4) a	A
C69	461.1656	7.29	Unknown	-	2.06	55.8 (± 4.4) a	37.4 (± 1.8) b	36.6 (± 0.9) b	K
C565	292.0903	31.30	Unknown	-	2.01	21.4 (± 3.5) b	32.5 (± 0.9) ab	34.1 (± 0.5) a	K
C408	487.1442	22.98	Unknown	-	1.95	35.7 (± 1.2) a	14.8 (± 3.5) b	13.5 (± 1.9) b	K
C18	167.0352	4.67	Homogentisate	Phenolic acid	1.95	31.9 (± 6.3) a	9.2 (± 1.3) b	9.9 (± 0.9) b	K
C99	471.1132	9.59	Compound WIN VIII	Dichlorobenzene	1.94	21.3 (± 5.1) a	7.8 (± 0.9) ab	5.1 (± 0.6) b	K
C506	592.0871	27.59	Unknown	-	1.91	22.6 (± 1.7) a	16.7 (± 0.7) ab	16.5 (± 0.3) b	K
C453	377.1242	24.88	Hydroxyvernolide	Sesquiterpenoids	1.86	68.3 (± 3.9) b	121.7 (± 6.5) a	113.1 (± 3.9) a	A
C452	575.1523	24.88	Unknown	-	1.86	27.6 (± 0.6) b	32.7 (± 0.5) a	32.6 (± 0.5) a	A
C545	711.1939	29.21	Bougainvillein-r-I	Betalain	1.83	22.0 (± 6.1) a	4.9 (± 0.3) b	4.8 (± 0.4) b	K
C352	955.2860	21.67	Unknown	-	1.82	18.0 (± 5.2) b	46.2 (± 1.9) a	44.5 (± 2.7) a	A
C179	431.1910	12.79	Unknown	-	1.81	26.1 (± 5.2) b	42.3 (± 1.1) a	41.9 (± 0.8) a	K
C120	191.0367	10.37	Scopoletin	Coumarins	1.80	25.8 (± 3.7) b	62.9 (± 6.5) a	70.4 (± 5.2) a	A
C360	477.1392	21.70	Unknown	-	1.78	225.3 (± 40.3) NS	330.6 (± 8.3) NS	338.6 (± 4.7) NS	K
C101	431.1556	9.73	Unknown	-	1.77	43.8 (± 7.1) a	27.2 (± 3.4) ab	17.9 (± 2.2) a	K
C348	475.1242	21.61	Unknown	-	1.71	91.4 (± 15.6) b	157.8 (± 10.9) a	151.5 (± 4.1) a	A
C400	621.1827	22.96	Orobanchoside	Caffeic acid ester	1.70	61.3 (± 11.4) a	38.6 (± 2.4) ab	32.0 (± 1.4) b	K
C431	563.1760	24.22	Pinocembrin 7-rhamnosylglucoside	Flavonoids	1.61	24.6 (± 2.7) a	10.0 (± 2.5) b	10.8 (± 1.5) b	A
C4	301.0921	2.16	Unknown	-	1.61	15.1 (± 0.9) a	12.3 (± 0.6) b	11.5 (± 0.4) b	A
C341	463.0878	21.57	Quercetin 3-O-glucoside	Flavonoids	1.61	39.5 (± 8.6) a	13.5 (± 1.9) b	15.8 (± 1.9) b	K
C409	623.1975	22.99	Forsythiaside	Organic acid	1.57	161.4 (± 25.8) a	123.7 (± 10.3) ab	91.6 (± 5.8) b	K
C169	369.0824	12.09	Fraxin	Coumarins	1.53	239.3 (± 18.4) b	275.2 (± 12.2) ab	309.6 (± 8.9) a	A
C436	447.0931	24.34	Quercitrin	Flavonoids	1.57	22.3 (± 5.6) b	32.9 (± 6.9) b	55.7 (± 4.5) a	K
C517	431.1328	28.68	Unknown	-	1.48	21.4 (± 4.9) a	8.9 (± 1.6) b	9.6 (± 0.9) b	K
C351	953.2707	21.67	Unknown	-	1.46	15.9 (± 3.6) b	37.3 (± 4.8) a	33.8 (± 1.9) a	A
C189	605.1519	12.98	Unknown	-	1.41	37.3 (± 7.2) NS	26.0 (± 4.7) NS	18.4 (± 1.7) NS	K
C473	461.1444	25.48	Unknown	-	1.37	18.2 (± 2.5) b	51. (± 6.0) a	41.6 (± 3.1) a	K
C267	613.2132	17.94	Unknown	-	1.35	30.1 (± 3.4) b	38.5 (± 1.9) ab	40.1 (± 1.4) a	K
C236	375.1447	14.94	Ailanthone	Triterpenoid	1.35	78.3 (± 4.9) a	49.7 (± 3.8) b	55.8 (± 3.5) b	K
C242	311.1130	15.81	4-Hydroxycinnamyl alcohol 4-D-glucoside	Monolignols	1.34	9.7 (± 2.8) b	14.9 (± 0.9) ab	18.4 (± 1.4) a	K
C586	289.1075	32.06	N-Succinyl-LL-2,6-diaminoheptanedioate	Organic acid	1.34	16.9 (± 2.2) a	9.3 (± 0.7) b	10.6 (± 0.9) b	A
C294	525.1602	19.52	Inumakilactone A glycoside	Diterpenoids	1.31	25.6 (± 6.2) a	17.3 (± 1.5) ab	14.3 (± 0.8) b	K
C379	279.0507	22.68	Unknown	-	1.29	49.6 (± 8.5) b	65.2 (± 5.1) a	67.4 (± 0.9) a	K
C171	737.1586	12.10	Unknown	-	1.29	16.1 (± 1.9) b	18.7 (± 1.3) ab	23.4 (± 1.3) a	A
C29	313.0925	5.08	Unknown	-	1.27	32.4 (± 5.0) b	41.2 (± 3.7) ab	46.4 (± 2.0) a	K
C380	473.1076	22.71	Unknown	-	1.26	27.9 (± 6.1) b	41.2 (± 6.3) ab	49.6 (± 3.4) a	K
C115	353.0865	10.23	Chlorogenate	Tannin	1.23	32.9 (± 1.0) b	43.5 (± 1.9) a	44.2 (± 2.4) a	K
C175	353.0865	10.23	Unknown	-	1.19	24.9 (± 1.1) b	26.9 (± 1.3) ab	29.9 (± 0.9) a	A
C82	732.0644	7.93	Unknown	-	1.18	20.7 (± 2.8) b	25.9 (± 1.8) ab	26.8 (± 0.7) a	K
C3	405.1389	2.05	Ipolamiide	Monoterpenes	1.18	25.4 (± 3.2) b	32.2 (± 1.5) ab	34.4 (± 1.7) a	K
C378	281.0657	22.68	Unknown	-	1.18	10.2 (± 0.6) c	30.0 (± 3.0) b	21.9 (± 1.9) a	A
C305	329.1391	20.24	Podolide	Diterpenoids	1.17	28.3 (± 5.0) NS	16.8 (± 1.8) NS	18.2 (± 1.4) NS	K
C617	284.0319	38.75	Riccionidin A	Flavonoids	1.15	25.6 (± 2.9) a	26.9 (± 5.8) a	37.9 (± 1.3) b	A
C172	192.0066	12.10	Unknown	-	1.11	26.5 (± 2.7) b	28.2 (± 1.6) ab	31.9 (± 0.9) a	A
C304	359.1497	20.24	Triptolide	Diterpenoids	1.07	36.8 (± 4.2) a	23.9 (± 2.5) b	26.1 (± 2.1) b	A
C235	537.1976	14.94	Unknown	-	1.04	74.8 (± 8.1) a	54.4 (± 5.3) ab	56.2 (± 3.6) b	A
C186	767.2056	12.98	Unknown	-	1.03	10.9 (± 2.4) a	13.5 (± 3.6) ab	4.2 (± 0.3) b	K
C317	507.1501	20.93	Unknown	-	1.02	19.7 (± 4.3) b	41.1 (± 6.9) a	39.9 (± 4.1) a	K
C411	505.2644	23.04	Unknown	-	1.02	8.6 (± 1.1) b	14.8 (± 1.5) a	15.5 (± 1.8) a	K
C165	369.8014	12.09	Unknown	-	1.01	10.8 (± 1.5) ab	10.9 (± 0.9) b	15.3 (± 1.1) a	A
C390	951.2548	22.80	Unknown	-	1.00	19.8 (± 0.8) b	30.2 (± 3.3) a	26.7 (± 1.1) a	K

*Different letters indicate a significant difference among the three groups as determined by Tukey’s HSD or Dunn’s test. NS = Not significant. * The letter refers to the type of statistical test applied: one-way ANOVA with Tukey´s HSF test (A) or Kruskal-Wallis with Dunn´s test (K).

Univariate analyses of metabolites abundance revealed that fifty-six compounds were statistically different between the three groups (Table 1). Within the flavonoids, ET had higher levels of quercitrin compared to ES, while AT showed the lowest amount. A similar pattern was observed for quercetrin 3-O-glucoside, where ET had slightly higher levels than ES, but AT exhibited the highest abundance. In contrast, riccionidin A was significantly higher in ET relative to both ES and AT. Two additional flavonoids, biochanin A 7-O-(6-O-malonyl-β-D-glucoside) and pinocembrin 7-rhamnosylglucoside, were found in significantly greater amounts in AT compared to both European groups. Among the coumarins, ET showed the highest levels of fraxetin and fraxin, followed by ES and then AT. For scopoletin, ET and ES displayed similar levels, both significantly higher than those in AT. Regarding iridoid glycosides, monotropein was detected at the highest amount in AT compared to either ET and ES.

### Leaf Tissue

A total of 381 features were detected with untargeted LC-MS analysis in the leaf samples (Fig. 1b). A full list of these features is presented in Table S2. Of these, approximately 65% of the features were shared among susceptible European ash (ES), tolerant European ash (ET) and Asian ash species (AT). PLS-DA distinguished Asian ash species (AT) but did not achieve a clear separation between susceptible European ash (ES) and tolerant European ash (ET) based on their metabolite profiles (Fig. 2b). PLS-DA revealed that the first component accounted for 15.8% and the second component accounted for 12.7% of the total variance and contributed to the best separation of the groups. The most important features were identified for the first PLS-DA component. Based on VIP scores, thirty-six features were identified as being important in explaining the differences between the three groups (Table 2). All 36 features were present in the three different groups, regardless of the missing value imputation. Based on their chemical families, compounds detected from ash leaf tissue included flavonoids, coumarins, iridoid glycosides, monolignol, phenolic glycoside (Fig. 3b).

**Table 2:** Mean (± standard error of the mean) ion counts (x10^2^) of the most informative chemical features in leaf tissue identified by identified by LC-MS/Q-TOF and PLS-DA analysis. Metabolites are listed in decreasing order of variable importance in the projection (VIP) scores

Feature number	[M-H]^–^(m/z)	Rt (min)	Putative ID	Biochemical class	VIP	AT	ES	ET	Statistical test*
C436	447.0931	24.34	Quercitrin	Flavonoids	2.29	69.3 (± 8.6) a	19.2 (± 2.3) b	27.8 (± 2.9) b	A
C290	373.1286	19.48	Kievitone hydrate	Flavonoids	2.17	8.9 (± 1.8) b	19.1 (± 2.5) a	25.1 (± 1.9) a	A
C435	285.0396	24.33	Luteolin	Flavonoids	2.04	22.7 (± 4.5) a	8.6 (± 1.7) b	9.0 (± 0.9) b	A
C89	337.0926	8.91	1-Caffeoyl-4-deoxyquinic acid	Organic acid	2.00	66.2 (± 12.9) a	14.3 (± 1.2) b	23.5 (± 3.5) b	K
C70	398.0294	7.31	Unknown	-	1.84	16.8 (± 0.9) b	19.1 (± 0.6) ab	19.9(± 0.4) a	K
C22	153.0558	4.95	Vanillyl alcohol	Alcohol	1.75	15.7 (± 2.9) b	25.0 (± 4.5) ab	32.8 (± 2.5) a	K
C147	241.0716	10.93	Lumichrome	Flavins	1.74	21.2 (± 2.3) b	31.7 (± 4.3) ab	41.8 (± 3.5) a	A
C113	215.0561	10.22	Unknown	-	1.71	27.8 (± 5.7) a	12.4 (± 2.6) ab	12.3 (1.8) b	K
C32	315.1084	5.08	Unknown	-	1.64	8.0 (± 1.9) b	19.3 (± 3.4) ab	29.5 (± 4.2) a	K
C633	480.2021	42.02	Unknown	-	1.60	72.6 (± 13.7) a	53.5 (± 13.3) ab	27.1 (± 6.5) b	K
C149	281.0662	11.09	4,4’-Diaminostilbene dihydrochloride	-	1.58	9.3 (± 1.3) b	18.1 (± 2.2) a	23.4 (± 2.9) a	K
C80	339.0718	7.87	Daphnin	Coumarins	1.55	36.0 (± 13.2) NS	13.5 (± 3.7) NS	11.6 (± 2.0) NS	K
C654	462.1916	47.75	Unknown	-	1.44	56.6 (± 11.9) a	46.4 (± 10.9) ab	23.9 (± 5.2) b	A
C208	197.0823	13.70	cis-2,3-Dihydroxy-2,3-dihydro-p-cumate	-	1.42	34.3 (± 3.9) b	69.6 (± 5.7) a	65.1 (± 6.4) a	K
C137	403.1243	10.83	Gardenoside	Iridoid glycoside	1.41	48.3 (± 9.5) NS	79.5 (± 10.7) NS	89.6 (± 8.5) NS	A
C67	119.0367	7.26	D-Erythrose	Aldoses	1.36	39.2 (± 3.1) b	50.4 (± 2.8) a	50.9 (± 2.5) a	K
C177	401.1444	12.58	Unknown	-	1.32	16.2 (± 5.6) NS	11.3 (± 4.4) NS	5.9 (± 0.7) NS	K
C114	351.0721	10.22	4-Methylumbelliferone glucuronide	Coumarins	1.32	74.9 (± 23.9) NS	30.1 (± 9.3) NS	33.9 (± 4.7) NS	K
C66	299.1137	7.26	Salidroside	Phenolic glycosides	1.27	31.6 (± 5.8) NS	42.8 (± 4.7) NS	59.2 (± 7.4) NS	K
C291	535.1817	19.48	Unknown	-	1.27	31.0 (± 4.2) b	44.9 (± 2.9) a	43.5 (± 2.6) a	K
C50	179.0358	6.58	3-(4-Hydroxyphenyl)pyruvate	-	1.27	38.1 (± 2.3) b	51.5 (± 3.5) a	51.2 (± 3.1) a	K
C533	569.1875	28.98	Decuroside III	Coumarins	1.23	17.2 (± 2.7) NS	29.5 (± 8.5) NS	48.0 (± 8.4) NS	K
C94	323.1336	9.30	Phaseollidin	Flavonoids	1.22	3.5 (± 0.8) NS	21.9 (± 5.5) NS	20.8 (± 4.2) NS	K
C419	593.1514	23.89	Isoorientin 2’’-O-rhamnoside	Flavonoids	1.19	90.6 (± 11.2) a	48.0 (± 5.3) b	58.9 (± 7.2) b	A
C534	223.0615	28.98	Sinapate	Monolignols	1.18	36.8 (± 2.2) NS	43.4 (± 5.9) NS	57.8 (± 6.0) NS	K
C337	461.0719	21.57	Luteolin 7-O-glucuronide	Flavonoids	1.18	37.1 (± 7.4) NS	26.2 (± 4.9) NS	23.2 (± 2.6) NS	K
C141	565.1774	10.83	Unknown	-	1.17	16.1 (± 3.7) NS	30.2 (± 4.9) NS	32.6 (± 4.4) NS	K
C84	165.0557	8.10	3-(2-Hydroxyphenyl)propanoate	-	1.16	6.9 (± 0.7) b	10.2 (± 1.5) ab	15.2 (± 2.5) a	K
C341	463.0878	21.57	Quercetin 3-O-glucoside	Flavonoids	1.11	69.8 (± 10.4) NS	43.9 (± 4.6) NS	48.4 (± 4.9) NS	A
C536	385.1138	28.99	1-O-Sinapoyl-beta-D-glucose	Monolignols	1.10	22.3 (± 1.9) NS	27.4 (± 6.3) NS	41.0 (± 5.7) NS	K
C144	367.1027	10.90	5-O-Feruloylquinic acid	-	1.10	38.5 (± 2.0) NS	35.9 (± 1.2) NS	31.2 (± 2.3) NS	K
C214	477.0689	13.71	Unknown	-	1.09	7.2 (± 0.5) NS	9.1 (± 0.8) NS	10.3 (± 0.9) NS	K
C479	220.0616	25.80	6-Hydroxyindolelactate	-	1.08	59.6 (± 5.3) NS	37.6 (± 6.1) NS	43.7 (± 3.5) NS	A
C126	345.1182	10.69	Aucubin	Iridoid glycoside	1.04	17.7 (± 1.3) NS	21.6 (± 1.7) NS	24.0 (± 2.1) NS	K
C61	652.1467	7.25	Unknown	-	1.01	10.4 (± 1.9) NS	12.3 (± 1.6) NS	14.7 (± 1.3) NS	A
C535	137.0605	28.99	3-Methoxybenzyl alcohol	-	1.00	10.9 (± 1.4) NS	14.9 (± 4.2) NS	22.6 (± 3.9) NS	K

*Different letters indicate a significant difference among the three groups as determined by Tukey’s HSD or Dunn’s test. NS = Not significant. * The letter refers to the type of statistical test applied: one-way ANOVA with Tukey´s HSF test (A) or Kruskal-Wallis with Dunn´s test (K)

Univariate analyses of metabolites abundance revealed that a limited proportion of features were statistically different between the three groups (Table 2). Among the flavonoids, ET and ES had slightly higher levels of kievitone hydrate and phaseollidin compared to AT. In contrast, AT had significantly higher amounts of quercitrin, luteolin, and isoorientin 2’’-O-rhamnoside than both ET and ES, while luteolin 7-O-glucuronide and quercetin 3-O-glucoside showed a similar, but non-significant, pattern. Three coumarins (daphnin, 4-methylumbelliferone glucuronide, and decuroside III) were detected, but had no significant differences among ET, ES, and AT. For iridoid glycosides, gardenoside and aucubin were more abundant in ET compared to ES and AT.

## Discussion

In this study, we analysed the constitutive soluble secondary metabolites in different genotypes of *F. excelsior* that differ in susceptibility to the ash dieback pathogen *H. fraxineus* and in three Asian ash species that are considered tolerant to the fungus. We recognized a total of 57 and 36 chemical features from phloem and leaf tissue, respectively, that differentiate the three phenotype groups (AT, ET, and ES). Flavonoid compounds composed a major portion of the secondary metabolites detected in the phloem and leaf samples. Flavonoids have been recorded as characteristic compounds for the genus *Fraxinus* (Kostova and Iossifova 2007) and play an important role in plant growth and development, as well as acting on plant defence mechanisms against biotic and abiotic factors in other plant systems (Wang et al. 2022; Witzell and Martín 2008). Among the five flavonoids identified in phloem tissues, tolerant European ash showed higher levels of quercitrin and quercitrin 3-O-glucoside than susceptible genotypes, while Asian ash had lower quercitrin but higher quercitrin 3-O-glucoside levels. In contrast, two quercitrin glycosides, isoqercitrin and rutin, were associated with susceptible European ash genotypes in the study by Nemesio-Gorriz et al. (2020). In leaves, seven flavonoids were detected, including quercitrin and quercitrin 3-O-glucoside, which were most abundant in Asian *Fraxinus*, followed by tolerant and then susceptible European ash. A study conducted on leaves of different ash species resistant and susceptible to emerald ash borer (EAB) found increased level of quercetrin 3-O-glucoside in resistant Manchurian ash and blue ash (*F. quadrangulata* Michx.) compared to the highly susceptible green (*F. pennsylvanica* Marshall) and black ash (*F. nigra* Marshall) (Qazi et al. 2018). Quercitrin is a prevalent flavonoid in many plants, contributing to seed germination, pollen production, photosynthesis, and overall growth and development (Singh et al. 2021). It has strong antioxidant properties, enhancing tolerance to abiotic stresses like heavy metals and UV light, as well as biotic stress from pests and pathogens (Singh et al. 2021; Treutter 2006). Quercitrin was found to inhibit herbivorous insect larvae growth and survival (Gao et al. 2022; Selin-Rani et al. 2016; Wang et al. 2019) and to suppress the growth of various bacteria, viruses, and fungi (Nguyen and Bhattacharya 2022). To date, no studies have examined the effect of quercitrin on *H. fraxineus*, and this gap needs be addressed before quercitrin can be considered as a suitable chemical biomarker. The flavones luteolin and luteolin 7-O-glucuronide were found only in leaf tissue and were more abundant in Asian ash species. Interestingly, these two flavonoids were detected only in susceptible American *Fraxinus* species but not in Manchurian ash in previous phytochemical studies on EAB resistance (Cipollini et al. 2011; Qazi et al. 2018; Whitehill et al. 2012).

In our study, we identified numerous flavonoids in their glycoside form, particularly at higher levels in Asian ash species. Glycosylation refers to the attachment of a sugar group to flavonoid compounds. This modification enhances their solubility and reduces their cytotoxicity, allowing them to be stored in plant tissues and mobilized when external stimuli occur, such as pathogen attack. The glycosides can then be acted upon by plant and fungal glycol to release aglycones (Kytidou et al. 2020; Ma et al. 2024). The higher level of flavonoid glycosides in Asian ash species may provide a larger pool of readily available bioactive compounds, acting as a crucial defensive mechanism that can be rapidly mobilized in response to *H. fraxineus*. This may explain why, as noted by Nielsen et al. (2017), Asian ash species can be hosts to the fungus but show milder symptoms than European and North American ash species, likely due to their defence response. All these findings, suggests that flavonoids and their glycosides derivative forms may play a key role in the defence responses of *Fraxinus* species to *H. fraxineus*. However, further research is needed to validate their role and mode of action in host susceptibility. Targeted metabolomics combined with functional assays, (i.e. pathogen challenge experiments measuring the rapid hydrolysis of flavonoid glycosides into aglycones, or genetic manipulation of glycosylation pathways) could provide direct evidence for whether these compounds contribute to tolerance in *Fraxinus* species.

The second class of metabolites that characterized our study are coumarins. Coumarins are a well-known and prevalent phytochemical in *Fraxinus* species and have been extensively studied for their potential role in the interaction between plants and bacteria, viruses, fungi and oomycetes (Kostova 2001; Kostova et al. 1993; Stringlis et al. 2019). In our study, three coumarins, tentatively identified as fraxetin, fraxin and, scopoletin, were detected in phloem tissue, which have also been reported in previous studies on *Fraxinus* spp. Fraxetin and its glucoside fraxin were detected in higher amount in tolerant European ash than in susceptible European and Asian ash species. These two coumarins have been reported by Nemesio-Gorriz et al. (2020), but only fraxetin was associated to tolerant genotypes. Moreover, fraxetin inhibited *H. fraxineus* growth in vitro when supplied at physiological concentrations, further supporting its potential as chemical biomarkers for ash dieback tolerance in European ash (Nemesio-Gorriz et al. 2020). In addition, scopoletin, a hydroxycoumarin, was identified in significantly higher amount from European ash genotypes in comparison to Asian ash species. Scopoletin was reported in significantly higher concentration from resistant blue ash in comparison to Manchurian ash and other susceptible American ash species by Qazi et al. (2018). The results of our study provide further support for the role of phloem coumarins and specifically fraxetin, in metabolite-based defence mechanisms.

Previous studies suggested the class of iridoid glycosides and secoiridoid glycosides as potential source for chemical biomarkers (Sambles et al. 2017; Sidda et al. 2020). However, the authors reported strong geographical effects on the ash glycosides composition, allowing *F. excelsior* genotypes to be distinguished based on their origin (UK or Danish) but not phenotype (tolerant or susceptible) (Sidda et al. 2020). In our study, only three iridoid glycosides were detected, and none of them were significantly different among the three groups. Only Asian ash species showed significantly increased levels of monotropein in phloem tissue. Although these iridoid glycosides were not significantly different in individual univariate analyses, they were still identified as important variables in the PLS-DA analysis, as shown by their VIP scores, suggesting that iridoid glycosides can contribute to host defence mechanisms but are not suitable for biomarkers identification.

Overall, the compounds discussed in this study tended to be present in higher amounts in tolerant European ash genotypes compared to susceptible ones, supporting hypothesis ii. In contrast, the phytochemical profiles of Asian ash species showed a more variable pattern, with some compounds present at higher levels and others at lower levels compared to European ash genotypes. Our study partially supported hypothesis i, as the variability in metabolites levels observed in Asian ash species is consistent with findings from other studies in the context of emerald ash borer. Previous studies have reported similar variability in metabolite levels, with certain compounds showing comparable concentrations between resistant Manchurian ash and susceptible American ash species (Cipollini et al. 2011; Eyles et al. 2007; Whitehill et al. 2012). Straight differences in metabolite amounts may not be sufficient to explain differences in phenotype, because while secondary metabolites play a crucial role in plant defence, other factors, including proteins and enzyme activities, may also contribute to resistance. For example, Cipollini et al. (2011) reported higher protein content and faster wound-browning in Manchurian ash phloem compared to highly susceptible American ash species, indicating a higher activity of phenol-oxidizing enzymes (Cipollini et al. 2011). Rigsby et al. (2015) and Rigsby et al. (2016) further suggested that the resistance of Manchurian ash might be due to the greater activities of oxidation and defence related enzymes. These results, indicate that several factors other than plant secondary metabolites are possibly involved in the resistance mechanisms of *Fraxinus* spp. particularly in tolerant Asian ash species (Rigsby et al. 2015, 2016). While our study did not assess enzymatic activity, it highlights the need for future investigations that integrate metabolomics with enzymatic assays to better understand tolerance mechanisms.

In this study we conducted a profiling of the constitutive metabolites in both tolerant and susceptible European ash genotypes as well as in three Asian ash species, analysing phloem and leaf tissue simultaneously. A more robust discrimination between the three groups was observed from phloem tissue. Unlike European ash, where *H. fraxineus* can extensively colonize woody tissue, the fungus shows restricted progression beyond leaves in its native Asian hosts (Nielsen et al. 2017). This difference in infection dynamics is thought to reflect the co-evolutionary relationship between the pathogen and its native hosts and suggests that the chemical composition of Asian ash phloem may play a role in limiting the spread and establishment of *H. fraxineus* in this tissue. However, it should be noted that due to the limited number of replicates per Asian ash species, we grouped all Asian ash species together based on their shared tolerance to *H. fraxineus* (Nielsen et al. 2017). This grouping enabled us to explore metabolite patterns associated with the tolerant phenotype, but may mask species-specific variation, which should be addressed in future studies with larger sample size. In addition, several of the constitutive metabolites detected in phloem tissue have previously been reported in studies on ash dieback and were also associated with resistance to EAB (Qazi et al. 2018; Villari et al. 2016; Whitehill et al. 2012), suggesting a potential cross-resistance between the two organisms that coexist in phloem tissue (Gossner et al. 2023). In a recent study on European ash, Gossner et al. (2023) identified the phenolic glycoside verbascoside as a key compound explaining variation in susceptibility to ADB and EAB. Verbascoside was constitutively present but showed a stronger induced response to EAB attack in tolerant genotypes, consistent with previous reports linking higher levels of this compound to greater EAB resistance (Whitehill et al. 2012, 2014). However, verbascoside was not detected in our untargeted approach. The absence of a compound in liquid chromatography-mass spectrometry analysis does not necessary indicate that it is not present in the samples. Instead, its non-detection could be due to various biological and technical factors, such as its concentration being below the detection limit of the mass spectrometer (Hrydziuszko and Viant 2012; Wei et al. 2018). Hence, we cannot exclude the role of this compound in *Fraxinus* spp. defence mechanisms. In addition, all the European ash genotypes of this study were selected within Sweden and geographical origin, environmental conditions, and seasonal factors are known to partially affecting the metabolite composition of plants (Sidda et al. 2020; Villari et al. 2018; Yang et al. 2018). Our sampling in June coincided with the period preceding fungal sporulation (Gross et al. 2014a, b; Hietala et al. 2013), making this time biologically relevant for capturing constitutive chemical profiles at the onset of host-pathogen interaction. Nevertheless, because the infection of woody tissue occurs later in the season, subsequent sampling of branch phloem may provide clearer picture on its defence chemistry. Thus, future research should focus on increasing the sampling material from diverse geographical origin and across different time points to obtain more comprehensive overview of European ash metabolite composition. Furthermore, in this study, we adopted an untargeted approach to screen all compounds present in the samples. Based on our results, a future objective could be to implement a targeted metabolites approach focusing on flavonoids and coumarins, allowing for higher selectivity and sensitivity in quantifying these compounds (Han et al. 2023; Lelli et al. 2021). Finally, in-vitro bioassays should be conducted on these compounds to provide further evidence of their potential role in ash defence mechanisms.

## Conclusions

In conclusion, this study has furthered our understanding of metabolite compositions of different *Fraxinus* species that differ in their co-evolutionary history and susceptibility to *H. fraxineus*. Our results indicate that flavonoids and coumarins, particularly fraxetin and quercitrin, are the primary compounds responsible for differentiating *Fraxinus* groups with varying susceptibility to ash dieback. While their presence is notable, their role in defence against ash dieback remains to be demonstrated. To deepen the understanding of defence mechanisms employed by *Fraxinus* spp., future studies should be targeted on flavonoids and coumarins. We then suggest that phloem tissue should be the primarily focus for further analyses for the identification of chemical biomarkers not only for ash dieback but also for emerald ash borer as both organisms coexist on this tissue.

## Supplementary Information

Below is the link to the electronic supplementary material.

Supplementary Material 1

## Data Availability

The data that support the findings of this study are available in the Metabolomics Workbench data repository, doi: http://dx.doi.org/10.21228/M8HZ7V.
