# Supplementary material for: Constitutive Metabolite Profiling of European and Asian Fraxinus with Varying Susceptibility to Ash Dieback
Source: J Chem Ecol. 2026 Jan 15;52(1):8. doi: 10.1007/s10886-025-01678-z (PMC12808147; doi:10.1007/s10886-025-01678-z)
Supplement: Supplementary file 1 — Supplementary Material 1 [file 10886_2025_1678_MOESM1_ESM.docx]

**Supplementary Information for:**

**Constitutive metabolite profiling of European and Asian *Fraxinus* with varying susceptibility to ash dieback**

Tolio, Beatrice^1,2^; Sherwood, Patrick^2^; Marčiulynienė, Diana^3^; Crocoll, Christoph^4^; Cleary, Michelle^2^; Liziniewicz, Mateusz^1^

*^1^* Skogforsk - The Forest Research Institute, Ekebo 2250, 268 90 Svalöv, Sweden

^2^ Southern Swedish Forest Research Centre, Swedish University of Agricultural Sciences, Sundsvägen 3, 234 56 Alnarp, Sweden

^3^ Institute of Forestry, Lithuanian Research Centre for Agriculture and Forestry, Liepų str. 1, Girionys, LT-53101 Kaunas district, Lithuania

^4^ Department of Plant and Environmental Sciences, University of Copenhagen, Thorvaldsensvej 40, 1871 Frederiksberg C, Denmark

Table S1 – List of the genotypes used in this study. For each genotype, species, susceptibility to ash dieback and the number of ramets sampled for each tissue type are provided.

| **Genotype ID** | **Species** | **Susceptibility to ash dieback*** | **No. of ramets** | |
| --- | --- | --- | --- | --- |
|  |  |  | Phloem | Leaves |
| 57 | *F. excelsior* | ET | 4 | 5 |
| 3003 | *F. excelsior* | ET | 3 | 3 |
| 3014 | *F. excelsior* | ET | 3 | 3 |
| 3019 | *F. excelsior* | ET | 2 | 2 |
| 3024 | *F. excelsior* | ET | 4 | 3 |
| 3026 | *F. excelsior* | ET | 2 | 2 |
| 3034 | *F. excelsior* | ET | 1 | 1 |
| 3045 | *F. excelsior* | ET | 1 | 1 |
| 3050 | *F. excelsior* | ET | 5 | 5 |
| 3001 | *F. excelsior* | ES | 4 | 4 |
| 3006 | *F. excelsior* | ES | 3 | 3 |
| 3025 | *F. excelsior* | ES | 2 | 2 |
| 3049 | *F. excelsior* | ES | 2 | 2 |
| 3052 | *F. chinensis* | AT | 2 | 2 |
| 3053 | *F. mandshurica* | AT | 3 | 3 |
| 3054 | *F. mandshurica* | AT | 1 | 1 |
| 3055 | *F. platypoda* | AT | 3 | 3 |
| 3056 | *F. platypoda* | AT | 1 | 1 |
|  |  |  | 46 | 46 |

* ET = European Tolerant, ES = European Susceptible, AT = Asian Tolerant

*Table S2 – List of all chemical features identified by LC-MS/Q-TOF in phloem (P) and leaf (L) tissue.*

| **[M-H]^–^(m/z)** | **RT (min)** | **Feature number** | **Putative ID** | **Tissue** |
| --- | --- | --- | --- | --- |
| 563.1398 | 18.58 | C1 | Schaftoside - Internal standard | L, P |
| 393.1386 | 2.02 | C2 | Rotenone | P |
| 405.1389 | 2.05 | C3 | Ipolamiide | P |
| 301.0921 | 2.16 | C4 | Unknown | P |
| 207.0872 | 2.18 | C5 | Unknown | L, P |
| 339.1291 | 2.18 | C6 | Methyl-2-alpha-L-fucopyranosyl-  beta-D-galactoside | L, P |
| 295.1027 | 2.32 | C7 | Unknown | L |
| 137.0239 | 2.72 | C8 | 4-Hydroxybenzoate | L |
| 393.1392 | 3.06 | C9 | Rotenone | L, P |
| 375.1286 | 3.81 | C10 | Riboflavin | L |
| 208.0979 | 3.94 | C11 | Tyr-OEt | L |
| 433.1341 | 4.09 | C12 | Unknown | P |
| 315.0717 | 4.10 | C13 | 2-(4-Chlorophenyl)-3-phenyl-3-(2-pyridinyl)acrylonitrile | L, P |
| 331.1030 | 4.23 | C14 | Tos-Arg-CH2Cl | P |
| 168.0428 | 4.23 | C15 | Phosphodimethylethanolamine | P |
| 379.0331 | 4.33 | C16 | Urate D-ribonucleotide | L, P |
| 329.0871 | 4.66 | C17 | 1-O-Vanilloyl-beta-D-glucose | P |
| 167.0352 | 4.67 | C18 | Homogentisate | L, P |
| 151.0397 | 4.77 | C19 | 4-Hydroxyphenylacetate | L, P |
| 313.0923 | 4.78 | C20 | Glucovanillin | L, P |
| 154.0590 | 4.95 | C21 | L-Histidine | P |
| 153.0558 | 4.95 | C22 | Vanillyl alcohol | L, P |
| 684.1362 | 5.05 | C23 | Unknown | L |
| 685.1401 | 5.05 | C24 | Unknown | P |
| 151.0399 | 5.06 | C25 | 4-Hydroxyphenylacetate | P |
| 632.2274 | 5.07 | C26 | Unknown | P |
| 314.0962 | 5.07 | C27 | Flusilazole | P |
| 317.1137 | 5.08 | C28 | Tebupirimfos | P |
| 313.0925 | 5.08 | C29 | Pyraclonil | P |
| 316.1120 | 5.08 | C30 | Unknown | L, P |
| 631.2244 | 5.08 | C31 | Unknown | P |
| 315.1084 | 5.08 | C32 | Etacelasil | L, P |
| 135.0448 | 5.09 | C33 | Phenyl acetate | L, P |
| 629.2080 | 5.09 | C34 | Unknown | P |
| 817.2419 | 5.12 | C35 | Unknown | P |
| 414.0243 | 5.13 | C36 | Unknown | L, P |
| 349.1495 | 5.27 | C37 | Propargite | P |
| 477.1603 | 5.56 | C38 | Unknown | P |
| 407.1551 | 6.08 | C39 | Devazepide | P |
| 389.1444 | 6.12 | C40 | Loganin | L, P |
| 313.1283 | 6.13 | C41 | Unknown | P |
| 197.0456 | 6.30 | C42 | 3-(3.4-Dihydroxyphenyl)lactate | P |
| 407.1548 | 6.42 | C43 | Devazepide | L, P |
| 389.1443 | 6.42 | C44 | Loganin | L, P |
| 354.0906 | 6.55 | C45 | Unknown | L |
| 177.0206 | 6.57 | C46 | 7.8-Dihydroxycoumarin | L |
| 351.0723 | 6.57 | C47 | 4-Methylumbelliferone glucuronide | L, P |
| 133.0291 | 6.57 | C48 | (S)-Ureidoglycolate | L, P |
| 353.0876 | 6.57 | C49 | Chlorogenate | L, P |
| 179.0358 | 6.58 | C50 | 3-(4-Hydroxyphenyl)pyruvate | L, P |
| 195.0298 | 6.58 | C51 | 3-(3.4-Dihydroxyphenyl)pyruvate | L, P |
| 409.0435 | 6.59 | C52 | Unknown | P |
| 713.1716 | 6.72 | C53 | Unknown | P |
| 167.0716 | 6.73 | C54 | N-Trimethyl-2-aminoethylphosphonate | P |
| 375.1285 | 6.73 | C55 | Riboflavin | P |
| 712.1682 | 6.73 | C56 | Guinea green B | P |
| 329.1241 | 6.74 | C57 | Unknown | P |
| 161.0456 | 6.74 | C58 | Lichenin | P |
| 398.0295 | 7.10 | C59 | Timepidium bromide | L, P |
| 300.1173 | 7.23 | C60 | Unknown | L, P |
| 652.1467 | 7.25 | C61 | Unknown | L, P |
| 301.1186 | 7.25 | C62 | Unknown | L, P |
| 599.2340 | 7.25 | C63 | Unknown | L, P |
| 149.0453 | 7.25 | C64 | D-Ribose | P |
| 653.1496 | 7.25 | C65 | Unknown | L, P |
| 299.1137 | 7.26 | C66 | Salidroside | L, P |
| 119.0367 | 7.26 | C67 | D-Erythrose | L, P |
| 345.1182 | 7.26 | C68 | Aucubin | L, P |
| 461.1656 | 7.29 | C69 | Unknown | L, P |
| 398.0294 | 7.31 | C70 | Timepidium bromide | L, P |
| 447.1494 | 7.45 | C71 | Unknown | P |
| 375.0475 | 7.70 | C72 | Unknown | L, P |
| 732.0637 | 7.75 | C73 | Unknown | L, P |
| 179.0253 | 7.86 | C74 | Unknown | P |
| 178.0238 | 7.86 | C75 | Unknown | P |
| 177.0207 | 7.86 | C76 | 7.8-Dihydroxycoumarin | L, P |
| 679.1511 | 7.87 | C77 | Unknown | P |
| 733.0678 | 7.87 | C78 | Unknown | P |
| 340.0758 | 7.87 | C79 | Unknown | L, P |
| 339.0718 | 7.87 | C80 | Daphnin | L, P |
| 341.0776 | 7.89 | C81 | Unknown | L, P |
| 732.0644 | 7.93 | C82 | Unknown | P |
| 483.9928 | 7.96 | C83 | Unknown | L, P |
| 165.0557 | 8.10 | C84 | 3-(2-Hydroxyphenyl)propanoate | L, P |
| 177.0546 | 8.37 | C85 | Vermelone | L |
| 295.0455 | 8.37 | C86 | Disulfiram | L |
| 164.0435 | 8.90 | C87 | L-Methionine S-oxide | L |
| 163.0404 | 8.91 | C88 | Phenylpyruvate | L, P |
| 337.0926 | 8.91 | C89 | 1-Caffeoyl-4-deoxyquinic acid | L, P |
| 179.0701 | 8.99 | C90 | Coniferyl alcohol | P |
| 329.1236 | 9.03 | C91 | Unknown | P |
| 443.1909 | 9.15 | C92 | Mitoxantrone | P |
| 457.1703 | 9.26 | C93 | Unknown | P |
| 323.1336 | 9.30 | C94 | Phaseollidin | L |
| 179.0360 | 9.33 | C95 | 3-(4-Hydroxyphenyl)pyruvate | L, P |
| 461.1658 | 9.49 | C96 | Unknown | L |
| 498.0084 | 9.57 | C97 | Unknown | P |
| 399.0924 | 9.58 | C98 | Unknown | P |
| 471.1132 | 9.59 | C99 | Compound WIN VIII | P |
| 325.0922 | 9.62 | C100 | 4-O-beta-D-Glucosyl-4-hydroxycinnamate | L, P |
| 431.1556 | 9.73 | C101 | Zizybeoside I | L, P |
| 432.1583 | 9.73 | C102 | Unknown | P |
| 567.1920 | 9.85 | C103 | Unknown | L, P |
| 505.1551 | 10.07 | C104 | Unknown | P |
| 445.1341 | 10.08 | C105 | Unknown | P |
| 707.1808 | 10.18 | C106 | Fe-coproporphyrin III | L, P |
| 703.1517 | 10.18 | C107 | Unknown | L, P |
| 369.0821 | 10.18 | C108 | Fraxin | L |
| 307.0812 | 10.19 | C109 | Allamandin | L |
| 705.1675 | 10.20 | C110 | Unknown | L, P |
| 173.0461 | 10.21 | C111 | Shikimate | L, P |
| 191.0545 | 10.21 | C112 | Quinate | L, P |
| 215.0561 | 10.22 | C113 | Terbacil | L, P |
| 351.0721 | 10.22 | C114 | 4-Methylumbelliferone glucuronide | L, P |
| 353.0865 | 10.23 | C115 | Chlorogenate | L, P |
| 352.0749 | 10.23 | C116 | Unknown | L, P |
| 760.0954 | 10.36 | C117 | Unknown | P |
| 192.0394 | 10.36 | C118 | Unknown | P |
| 176.0122 | 10.36 | C119 | Unknown | P |
| 191.0367 | 10.37 | C120 | Scopoletin | L, P |
| 531.1344 | 10.49 | C121 | Trichotomine | P |
| 209.0817 | 10.54 | C122 | Sinapyl alcohol | P |
| 135.0297 | 10.59 | C123 | Hypoxanthine | L, P |
| 329.1240 | 10.65 | C124 | Unknown | P |
| 390.1113 | 10.68 | C125 | Unknown | L, P |
| 345.1182 | 10.69 | C126 | Aucubin | L, P |
| 389.1086 | 10.69 | C127 | Monotropein | L, P |
| 209.0454 | 10.70 | C128 | 5-Hydroxyferulic acid | L |
| 183.0667 | 10.70 | C129 | Choline phosphate | L, P |
| 603.1508 | 10.80 | C130 | Unknown | L, P |
| 566.1796 | 10.81 | C131 | Unknown | L, P |
| 404.1275 | 10.82 | C132 | Unknown | L, P |
| 618.0876 | 10.83 | C133 | ADP-D-glycero-beta-D-manno-heptose | L, P |
| 601.1532 | 10.83 | C134 | Amaroswerin | L, P |
| 405.1286 | 10.83 | C135 | Unknown | L, P |
| 611.1825 | 10.83 | C136 | Unknown | L, P |
| 403.1243 | 10.83 | C137 | Gardenoside | L, P |
| 445.1337 | 10.83 | C138 | Unknown | L, P |
| 612.1853 | 10.83 | C139 | Unknown | L, P |
| 789.2461 | 10.83 | C140 | Unknown | L, P |
| 565.1774 | 10.83 | C141 | Unknown | L, P |
| 951.2988 | 10.83 | C142 | Unknown | L |
| 505.1550 | 10.83 | C143 | Unknown | L |
| 367.1027 | 10.90 | C144 | 5-O-Feruloylquinic acid | L, P |
| 193.0506 | 10.92 | C145 | Scytalone | L, P |
| 449.1286 | 10.93 | C146 | Unknown | L, P |
| 241.0716 | 10.93 | C147 | Lumichrome | L, P |
| 545.1498 | 10.95 | C148 | Xanthoaphin | P |
| 281.0662 | 11.09 | C149 | 4.4'-Diaminostilbene dihydrochloride | L |
| 349.1858 | 11.27 | C150 | Phenothrin | P |
| 173.0460 | 11.28 | C151 | Shikimate | P |
| 207.0304 | 11.50 | C152 | Fraxetin | L, P |
| 481.2275 | 11.78 | C153 | Unknown | P |
| 401.1446 | 11.87 | C154 | Unknown | L, P |
| 501.1238 | 11.96 | C155 | Fluvalinate | P |
| 206.0225 | 11.98 | C156 | p-Aminobenzamidine dihydrochloride | P |
| 223.0595 | 12.01 | C157 | Sinapate | L, P |
| 503.0482 | 12.05 | C158 |  | L |
| 368.9990 | 12.07 | C159 | Sedoheptulose 1.7-bisphosphate | P |
| 372.0891 | 12.07 | C160 | Deacetylcephalosporin C | P |
| 208.0337 | 12.08 | C161 | N-Acetyldemethylphosphinothricin | P |
| 370.0863 | 12.08 | C162 | Berberine chloride | L, P |
| 371.0891 | 12.08 | C163 | Ohioensin-A | L, P |
| 369.6607 | 12.09 | C164 | Unknown | P |
| 369.8014 | 12.09 | C165 | Unknown | P |
| 207.0305 | 12.09 | C166 | Fraxetin | L, P |
| 354.0585 | 12.09 | C167 | Unknown | P |
| 738.1610 | 12.09 | C168 | Unknown | P |
| 369.0824 | 12.09 | C169 | Fraxin | L, P |
| 369.5994 | 12.10 | C170 | Unknown | P |
| 737.1586 | 12.10 | C171 | Unknown | P |
| 192.0066 | 12.10 | C172 | Unknown | P |
| 207.0304 | 12.12 | C173 | Fraxetin | L, P |
| 793.0895 | 12.19 | C174 | Unknown | P |
| 792.0863 | 12.22 | C175 | Unknown | L, P |
| 269.1024 | 12.57 | C176 | Chloroprocaine | L, P |
| 401.1444 | 12.58 | C177 | Unknown | L, P |
| 385.1858 | 12.78 | C178 | Unknown | L, P |
| 431.1910 | 12.79 | C179 | Aspulvinone H | L, P |
| 528.0191 | 12.89 | C180 | Unknown | P |
| 820.1175 | 12.95 | C181 | Propenoyl-CoA | P |
| 431.1082 | 12.96 | C182 | Unknown | P |
| 384.1010 | 12.96 | C183 | S-Inosyl-L-homocysteine | P |
| 222.0496 | 12.97 | C184 | Selfotel | L, P |
| 223.0509 | 12.98 | C185 | Nifuradene | P |
| 767.2056 | 12.98 | C186 | Unknown | P |
| 606.1542 | 12.98 | C187 | Unknown | P |
| 430.1067 | 12.98 | C188 | Unknown | P |
| 605.1519 | 12.98 | C189 | Unknown | P |
| 383.0984 | 12.99 | C190 | Unknown | P |
| 190.9989 | 12.99 | C191 | Unknown | P |
| 429.1040 | 12.99 | C192 | Bispyribac | L, P |
| 206.0224 | 12.99 | C193 | p-Aminobenzamidine dihydrochloride | P |
| 221.0458 | 12.99 | C194 | 2-Succinylbenzoate | L, P |
| 207.0370 | 13.00 | C195 | 2-(2'-Methylthio)ethylmalic acid | P |
| 528.0193 | 13.11 | C196 | Unknown | P |
| 383.1436 | 13.14 | C197 | Unknown | L |
| 309.1084 | 13.16 | C198 | 7-Hydroxy-3-(4-methoxyphenyl)-4-propyl-2LH-1-benzopyran-2-one | L |
| 225.0766 | 13.19 | C199 | Genipin | L, P |
| 433.1337 | 13.19 | C200 | Unknown | L, P |
| 193.0504 | 13.25 | C201 | Scytalone | L |
| 467.9777 | 13.34 | C202 | Unknown | P |
| 387.1657 | 13.42 | C203 | Tuberonic acid glucoside | L, P |
| 515.1393 | 13.54 | C204 | Unknown | P |
| 341.1235 | 13.54 | C205 | Coniferin | L, P |
| 281.1022 | 13.54 | C206 | 2-Aminoadenosine | P |
| 433.2067 | 13.69 | C207 | Flunisolide | P |
| 197.0823 | 13.70 | C208 | cis-2.3-Dihydroxy-2.3-dihydro-p-cumate | L, P |
| 378.1474 | 13.70 | C209 | Kasugamycin | L |
| 377.1448 | 13.70 | C210 | Reduced riboflavin | L, P |
| 198.0853 | 13.70 | C211 | gamma-Glutamyl-beta-aminopropiononitrile | L |
| 153.0918 | 13.70 | C212 | Boschnialactone | L |
| 207.0490 | 13.71 | C213 | Dihydrolipoate | P |
| 477.0689 | 13.71 | C214 | Unknown | L |
| 215.0924 | 13.75 | C215 | Unknown | L |
| 517.2282 | 13.75 | C216 | Unknown | L, P |
| 179.0713 | 13.88 | C217 | Coniferyl alcohol | L, P |
| 583.2028 | 13.89 | C218 | Unknown | L, P |
| 375.1441 | 13.89 | C219 | Ailanthone | L, P |
| 357.1182 | 13.89 | C220 | Tarennoside | L, P |
| 537.1965 | 13.89 | C221 | Unknown | L |
| 323.0976 | 14.04 | C222 | Bis-D-fructose 2'.1:2.1'-dianhydride | P |
| 541.1554 | 14.21 | C223 | Unknown | P |
| 333.0611 | 14.64 | C224 | sn-Glycero-3-phospho-1-inositol | L, P |
| 179.0358 | 14.64 | C225 | 3-(4-Hydroxyphenyl)pyruvate | L, P |
| 335.0769 | 14.64 | C226 | Altersolanol A | L, P |
| 336.0800 | 14.65 | C227 | Unknown | L, P |
| 519.2435 | 14.78 | C228 | Unknown | P |
| 583.2033 | 14.82 | C229 | Unknown | P |
| 327.1089 | 14.82 | C230 | Anisatin | P |
| 489.1599 | 14.82 | C231 | Unknown | P |
| 179.0717 | 14.93 | C232 | Coniferyl alcohol | P |
| 538.2001 | 14.93 | C233 | Unknown | P |
| 195.0661 | 14.93 | C234 | 2.3-Dihydroxy-p-cumate | P |
| 537.1976 | 14.94 | C235 | Unknown | P |
| 375.1447 | 14.94 | C236 | Ailanthone | P |
| 376.1474 | 14.94 | C237 | Imidaprilat | P |
| 573.1730 | 14.94 | C238 | Unknown | P |
| 495.1496 | 15.44 | C239 | Mycophenolic acid O-acyl-glucuronide | P |
| 191.0566 | 15.56 | C240 | Quinate | P |
| 367.1026 | 15.57 | C241 | 5-O-Feruloylquinic acid | L, P |
| 311.1130 | 15.81 | C242 | 4-Hydroxycinnamyl alcohol 4-D-glucoside | P |
| 416.1630 | 15.99 | C243 | Unknown | L, P |
| 415.1602 | 16.00 | C244 | Unknown | L, P |
| 149.0453 | 16.01 | C245 | D-Ribose | L, P |
| 385.0000 | 16.09 | C246 | Chlorimuron | P |
| 371.0973 | 16.10 | C247 | Ohioensin-A | L, P |
| 383.9995 | 16.10 | C248 | Unknown | L, P |
| 493.1336 | 16.56 | C249 | Unknown | P |
| 393.1756 | 16.56 | C250 | Unknown | L |
| 384.1289 | 16.62 | C251 | Prifinium bromide | L |
| 305.0695 | 16.81 | C252 | Leucocyanidin | L |
| 525.1601 | 16.84 | C253 | Inumakilactone A glycoside | P |
| 493.1339 | 16.87 | C254 | Unknown | P |
| 238.0657 | 16.96 | C255 | Clomazone | P |
| 333.1548 | 16.99 | C256 | Veprisinium | L |
| 251.0553 | 17.24 | C257 | S-(4-Methylthiobutylthiohydroximoyl)-L-cysteine | P |
| 734.1513 | 17.56 | C258 | Unknown | P |
| 717.2164 | 17.57 | C259 | Unknown | P |
| 727.2457 | 17.57 | C260 | Unknown | P |
| 681.2412 | 17.58 | C261 | Bruceoside A | P |
| 347.1706 | 17.60 | C262 | Schizonepetoside E | L |
| 537.1963 | 17.64 | C263 | Unknown | L |
| 359.1340 | 17.82 | C264 | 7-Deoxyloganate | L, P |
| 637.1765 | 17.88 | C265 | Unknown | P |
| 639.1931 | 17.92 | C266 | Plantamajoside | P |
| 613.2132 | 17.94 | C267 | Unknown | P |
| 405.1544 | 17.94 | C268 | Unknown | P |
| 311.0944 | 18.02 | C269 | Vicianose | P |
| 639.1933 | 18.21 | C270 | Plantamajoside | L |
| 319.0816 | 18.38 | C271 | 4-Coumaroylshikimate | L, P |
| 535.2746 | 18.51 | C272 | Unknown | L, P |
| 711.2508 | 18.60 | C273 | Unknown | P |
| 639.1927 | 18.62 | C274 | Plantamajoside | P |
| 185.0458 | 18.68 | C275 | Unknown | L |
| 417.1388 | 18.68 | C276 | Cyclomorusin | L |
| 255.0871 | 18.69 | C277 | Phenylgalactoside | L, P |
| 463.1448 | 18.69 | C278 | Unknown | L, P |
| 562.0612 | 18.70 | C279 | Unknown | L |
| 521.2019 | 18.96 | C280 | Isobrucein A | L, P |
| 361.1655 | 19.03 | C281 | Gibberellin A19 | P |
| 523.2179 | 19.03 | C282 | Mascaroside | P |
| 524.2201 | 19.04 | C283 | Unknown | P |
| 392.1201 | 19.22 | C284 | Tifluadom | P |
| 785.2527 | 19.22 | C285 | Echinacoside | P |
| 374.1314 | 19.47 | C286 | Haloperidol | L, P |
| 571.1573 | 19.47 | C287 | Unknown | P |
| 211.0611 | 19.47 | C288 | Danielone | P |
| 581.1872 | 19.48 | C289 | Unknown | L, P |
| 373.1286 | 19.48 | C290 | Kievitone hydrate | L, P |
| 535.1817 | 19.48 | C291 | Unknown | L, P |
| 343.1173 | 19.48 | C292 | Calaxin | P |
| 536.1843 | 19.49 | C293 | Unknown | L, P |
| 525.1602 | 19.52 | C294 | Inumakilactone A glycoside | L, P |
| 403.1380 | 19.53 | C295 | Mallotophenone | P |
| 787.2674 | 19.59 | C296 | Phomopsin A | P |
| 741.2619 | 19.60 | C297 | Acanthoside D | P |
| 579.2081 | 19.60 | C298 | (+)-Syringaresinol O-beta-D-glucoside | P |
| 185.1185 | 19.71 | C299 | 10-Oxodecanoate | L |
| 551.1403 | 19.81 | C300 | Unknown | L, P |
| 360.1527 | 20.24 | C301 | Anisotropine methylbromide | P |
| 522.2049 | 20.24 | C302 | Unknown | P |
| 521.2025 | 20.24 | C303 | Isobrucein A | P |
| 359.1497 | 20.24 | C304 | Triptolide | P |
| 329.1391 | 20.24 | C305 | Podolide | P |
| 503.2475 | 20.32 | C306 | Unknown | P |
| 637.1767 | 20.47 | C307 | Unknown | L, P |
| 567.2018 | 20.50 | C308 | Unknown | P |
| 327.1227 | 20.52 | C309 | Decursin | P |
| 566.1943 | 20.53 | C310 | Unknown | P |
| 565.1921 | 20.53 | C311 | Unknown | L, P |
| 611.2544 | 20.59 | C312 | Unknown | L, P |
| 403.1377 | 20.87 | C313 | Mallotophenone | P |
| 707.1834 | 20.91 | C314 | Fe-coproporphyrin III | P |
| 505.1337 | 20.91 | C315 | Streptonigrin | P |
| 508.1533 | 20.92 | C316 | Unknown | P |
| 507.1501 | 20.93 | C317 | Unknown | P |
| 463.2169 | 21.08 | C318 | Unknown | P |
| 293.0874 | 21.09 | C319 | Tuliposide B | P |
| 443.1551 | 21.11 | C320 | Fluticasone | P |
| 444.1579 | 21.12 | C321 | Unknown | P |
| 625.1395 | 21.16 | C322 | Quercetin 3-O-beta-D-glucosyl-(1->2)-beta-D-glucoside | L |
| 149.0600 | 21.16 | C323 | Tolylacetate | L, P |
| 611.1513 | 21.16 | C324 | Unknown | L, P |
| 610.1496 | 21.16 | C325 | Cyanidin 3.5-O-diglucoside | L, P |
| 727.0805 | 21.16 | C326 | Unknown | L |
| 608.1328 | 21.16 | C327 | Unknown | L |
| 612.1530 | 21.16 | C328 | Unknown | L |
| 609.1460 | 21.16 | C329 | Rutin | L, P |
| 607.1301 | 21.17 | C330 | Unknown | L, P |
| 503.2480 | 21.22 | C331 | Unknown | P |
| 581.1498 | 21.28 | C332 | Asperuloside tetraacetate | P |
| 195.0298 | 21.38 | C333 | 3-(3.4-Dihydroxyphenyl)pyruvate | P |
| 323.0767 | 21.39 | C334 | Unknown | P |
| 595.2021 | 21.45 | C335 | Unknown | P |
| 464.0910 | 21.56 | C336 | Unknown | L, P |
| 461.0719 | 21.57 | C337 | Luteolin 7-O-glucuronide | L, P |
| 927.1842 | 21.57 | C338 | Unknown | L |
| 462.0746 | 21.57 | C339 | Unknown | L, P |
| 301.0340 | 21.57 | C340 | Quercetin | L, P |
| 463.0878 | 21.57 | C341 | Quercetin 3-O-glucoside | L, P |
| 341.0874 | 21.57 | C342 | Caffeic acid 3-glucoside | P |
| 465.0921 | 21.57 | C343 | Unknown | L, P |
| 479.0815 | 21.58 | C344 | Gossypetin 8-O-glucoside | L, P |
| 301.0345 | 21.59 | C345 | Quercetin | L |
| 941.2325 | 21.59 | C346 | Unknown | P |
| 476.1276 | 21.59 | C347 | Unknown | P |
| 475.1242 | 21.61 | C348 | Unknown | L, P |
| 954.2738 | 21.66 | C349 | Unknown | P |
| 951.2555 | 21.66 | C350 | Unknown | P |
| 953.2707 | 21.67 | C351 | Unknown | P |
| 955.2860 | 21.67 | C352 | Unknown | P |
| 956.2893 | 21.68 | C353 | Unknown | P |
| 478.4261 | 21.68 | C354 | Unknown | P |
| 478.1431 | 21.68 | C355 | S-[(E)-N-Hydroxy(indol-3-yl)acetimidoyl]-L-glutathione | L, P |
| 479.1460 | 21.68 | C356 | Blestriarene B | P |
| 480.1488 | 21.68 | C357 | Unknown | P |
| 477.0418 | 21.68 | C358 | Fucofuroeckol B | P |
| 477.9535 | 21.69 | C359 | Unknown | P |
| 477.1392 | 21.70 | C360 | Unknown | L, P |
| 555.1719 | 21.77 | C361 | 7-Dehydrologanin tetraacetate | L, P |
| 556.1745 | 21.77 | C362 | Unknown | L, P |
| 393.1183 | 21.78 | C363 | Aloesin | L, P |
| 663.1556 | 21.85 | C364 | Unknown | P |
| 427.1268 | 21.87 | C365 | Unknown | L |
| 652.1952 | 21.90 | C366 | Unknown | L |
| 651.1925 | 21.91 | C367 | Esmeraldin B | L, P |
| 653.2093 | 21.93 | C368 | Unknown | L, P |
| 654.2119 | 21.93 | C369 | Unknown | L, P |
| 706.1197 | 21.93 | C370 | Unknown | L |
| 655.2140 | 21.93 | C371 | Unknown | L, P |
| 433.0760 | 22.44 | C372 | Unknown | L, P |
| 593.1511 | 22.47 | C373 | Isoorientin 2''-O-rhamnoside | P |
| 474.1108 | 22.59 | C374 | Unknown | P |
| 323.0763 | 22.60 | C375 | Unknown | P |
| 251.0557 | 22.63 | C376 | S-(4-Methylthiobutylthiohydroximoyl)-L-cysteine | P |
| 341.0874 | 22.67 | C377 | Caffeic acid 3-glucoside | P |
| 281.0657 | 22.68 | C378 | 4.4'-Diaminostilbene dihydrochloride | P |
| 279.0507 | 22.68 | C379 | Sulfamonomethoxine | P |
| 473.1076 | 22.71 | C380 | Unknown | P |
| 480.1493 | 22.78 | C381 | Unknown | P |
| 477.1397 | 22.78 | C382 | Unknown | P |
| 477.7969 | 22.78 | C383 | Unknown | P |
| 952.2586 | 22.78 | C384 | Unknown | P |
| 487.1440 | 22.79 | C385 | Unknown | L, P |
| 478.4259 | 22.79 | C386 | Unknown | P |
| 477.0407 | 22.79 | C387 | Unknown | P |
| 479.1461 | 22.79 | C388 | Blestriarene B | P |
| 477.9534 | 22.79 | C389 | Unknown | P |
| 951.2548 | 22.80 | C390 | Unknown | P |
| 161.0265 | 22.81 | C391 | Umbelliferone | P |
| 509.1655 | 22.87 | C392 | Unknown | L, P |
| 357.1341 | 22.89 | C393 | (+)-Pinoresinol | L, P |
| 521.1928 | 22.90 | C394 | Unknown | P |
| 519.1867 | 22.90 | C395 | Brusatol | L, P |
| 359.1399 | 22.90 | C396 | Unknown | P |
| 161.0434 | 22.91 | C397 | Lichenin | P |
| 151.0400 | 22.91 | C398 | 4-Hydroxyphenylacetate | P |
| 622.1855 | 22.95 | C399 | Unknown | L, P |
| 621.1827 | 22.96 | C400 | Orobanchoside | L, P |
| 620.1685 | 22.97 | C401 | Unknown | L, P |
| 626.2067 | 22.97 | C402 | Unknown | L, P |
| 625.2046 | 22.97 | C403 | Unknown | L, P |
| 741.1326 | 22.98 | C404 | Unknown | L, P |
| 619.1657 | 22.98 | C405 | Unknown | L, P |
| 624.2016 | 22.98 | C406 | Unknown | L, P |
| 624.1017 | 22.98 | C407 | Unknown | L, P |
| 487.1442 | 22.98 | C408 | Unknown | L, P |
| 623.1975 | 22.99 | C409 | Forsythiaside | L, P |
| 551.2696 | 23.03 | C410 | Canthiumine | P |
| 505.2644 | 23.04 | C411 | Unknown | P |
| 676.1086 | 23.07 | C412 | Unknown | L |
| 701.2301 | 23.15 | C413 | Unknown | L, P |
| 323.0767 | 23.32 | C414 | Unknown | P |
| 293.0872 | 23.71 | C415 | Tuliposide B | L, P |
| 596.1578 | 23.88 | C416 | Unknown | L |
| 595.1563 | 23.88 | C417 | Unknown | L, P |
| 594.1536 | 23.88 | C418 | Unknown | L, P |
| 593.1514 | 23.89 | C419 | Isoorientin 2''-O-rhamnoside | L, P |
| 531.1131 | 23.89 | C420 | Biochanin A 7-O-(6-O-malonyl-beta-D-glucoside) | P |
| 503.0811 | 23.95 | C421 | Hypericin | L |
| 505.0969 | 23.95 | C422 | Cassiamin C | L |
| 725.1942 | 23.97 | C423 | Unknown | L, P |
| 369.1326 | 24.05 | C424 | 5'-Prenylhomoeriodictyol | P |
| 550.2001 | 24.05 | C425 | Unknown | P |
| 387.1448 | 24.05 | C426 | Cetirizine | L, P |
| 388.1478 | 24.05 | C427 | Unknown | P |
| 549.1975 | 24.06 | C428 | Eucommin A | P |
| 418.1573 | 24.17 | C429 | Unknown | P |
| 417.1552 | 24.18 | C430 | Euparotin acetate | P |
| 563.1760 | 24.22 | C431 | Pinocembrin 7-rhamnosylglucoside | P |
| 339.1229 | 24.28 | C432 | Glepidotin B | L, P |
| 448.0960 | 24.33 | C433 | Cyhalothrin | L, P |
| 449.0973 | 24.33 | C434 | Unknown | L, P |
| 285.0396 | 24.33 | C435 | Luteolin | L, P |
| 447.0931 | 24.34 | C436 | Quercitrin | L, P |
| 741.1340 | 24.42 | C437 | Unknown | P |
| 619.1666 | 24.43 | C438 | Unknown | P |
| 701.2301 | 24.43 | C439 | Unknown | P |
| 676.1096 | 24.45 | C440 | Unknown | P |
| 492.2012 | 24.49 | C441 | Unknown | L |
| 623.1810 | 24.66 | C442 | Unknown | P |
| 624.1697 | 24.67 | C443 | Unknown | L, P |
| 623.1670 | 24.68 | C444 | Zinc protoporphyrin-9 | L, P |
| 499.0873 | 24.71 | C445 | Unknown | P |
| 501.1030 | 24.72 | C446 | Malonyldaidzin | P |
| 379.1290 | 24.86 | C447 | Unknown | L, P |
| 378.1276 | 24.87 | C448 | Unknown | L, P |
| 359.1126 | 24.87 | C449 | Acalyphin | L, P |
| 638.0932 | 24.87 | C450 | Unknown | L, P |
| 539.1766 | 24.88 | C451 | Oleuropein | L, P |
| 575.1523 | 24.88 | C452 | Unknown | L, P |
| 377.1242 | 24.88 | C453 | Hydroxyvernolide | L, P |
| 419.1198 | 24.88 | C454 | Secogalioside | L, P |
| 755.2043 | 24.91 | C455 | Unknown | P |
| 581.1501 | 25.00 | C456 | Asperuloside tetraacetate | L, P |
| 522.1968 | 25.01 | C457 | Deacetylisoipecoside | L |
| 360.1446 | 25.03 | C458 | 3-Acetylnerbowdine | L |
| 477.1051 | 25.15 | C459 | Chlortetracycline | L, P |
| 478.1089 | 25.16 | C460 | Petunidin 3-O-glucoside | P |
| 547.1655 | 25.29 | C461 | Unknown | P |
| 769.2208 | 25.29 | C462 | Unknown | P |
| 731.2403 | 25.30 | C463 | Unknown | L, P |
| 687.2402 | 25.30 | C464 | Unknown | L |
| 685.2361 | 25.30 | C465 | Unknown | L, P |
| 686.2387 | 25.31 | C466 | Unknown | L, P |
| 721.2114 | 25.31 | C467 | Unknown | L, P |
| 489.1388 | 25.31 | C468 | Unknown | P |
| 491.1551 | 25.32 | C469 | Unknown | P |
| 607.2027 | 25.43 | C470 | Unknown | L, P |
| 459.1281 | 25.47 | C471 | Unknown | P |
| 462.1476 | 25.48 | C472 | Unknown | P |
| 461.1444 | 25.48 | C473 | Unknown | L, P |
| 519.1496 | 25.54 | C474 | Chryso-obtusin glucoside | P |
| 569.1876 | 25.75 | C475 | Decuroside III | P |
| 607.2033 | 25.76 | C476 | Unknown | L |
| 407.1335 | 25.76 | C477 | Amlodipine | P |
| 340.1183 | 25.79 | C478 | Cassythine | L |
| 220.0616 | 25.80 | C479 | 6-Hydroxyindolelactate | L |
| 489.1862 | 25.98 | C480 | Unknown | L |
| 573.2182 | 26.11 | C481 | Unknown | L, P |
| 709.1990 | 26.25 | C482 | Unknown | L |
| 755.2061 | 26.26 | C483 | Unknown | L, P |
| 633.2395 | 26.39 | C484 | Unknown | P |
| 638.2164 | 26.52 | C485 | Unknown | P |
| 635.1974 | 26.52 | C486 | Unknown | P |
| 637.2142 | 26.52 | C487 | Unknown | L, P |
| 527.2119 | 26.74 | C488 | Karwinskione | L, P |
| 491.1556 | 26.96 | C489 | Unknown | P |
| 429.1753 | 27.12 | C490 | 2.2-Dimethyl-3.4-bis(4-methoxyphenyl)-2H-1-benzopyran-7-ol acetate | L, P |
| 249.1125 | 27.12 | C491 | Diisopropyl phthalate | P |
| 489.1028 | 27.23 | C492 | FMN-N5-peroxide | L |
| 461.1080 | 27.24 | C493 | Isoscoparine | P |
| 547.1804 | 27.36 | C494 | Flavanone 7-O-[alpha-L-rhamnosyl-(1->2)-beta-D-glucoside] | P |
| 592.0873 | 27.38 | C495 | Unknown | L, P |
| 116.9282 | 27.46 | C496 | Unknown | P |
| 540.1799 | 27.48 | C497 | Unknown | L, P |
| 542.1836 | 27.49 | C498 | Unknown | L, P |
| 541.1826 | 27.49 | C499 | Unknown | L, P |
| 540.0432 | 27.49 | C500 | Unknown | P |
| 539.1763 | 27.50 | C501 | Oleuropein | L, P |
| 345.0970 | 27.50 | C502 | (6R)-2-Acetyl-6-(3-acetyl-2.4.6-trihydroxy-5-methylphenyl)-3-hydroxy-6-methyl-2.4-cyclohexadien-1-one | L, P |
| 275.0920 | 27.50 | C503 | Glutamyl-glutamic acid | L, P |
| 307.0819 | 27.51 | C504 | Allamandin | L, P |
| 377.1238 | 27.51 | C505 | Hydroxyvernolide | L, P |
| 592.0871 | 27.59 | C506 | Unknown | L, P |
| 337.1071 | 27.85 | C507 | (-)-Glyceollin I | P |
| 505.1701 | 28.15 | C508 | Unknown | P |
| 265.0746 | 28.23 | C509 | Ungeremine | L |
| 575.1399 | 28.27 | C510 | Unknown | P |
| 503.1541 | 28.30 | C511 | Sergeolide | L, P |
| 746.2755 | 28.46 | C512 | Unknown | P |
| 745.2726 | 28.47 | C513 | Unknown | P |
| 485.1076 | 28.53 | C514 | Flavonol 3-O-(6-O-malonyl-beta-D-glucoside) | P |
| 545.1294 | 28.53 | C515 | Unknown | P |
| 275.0918 | 28.54 | C516 | Glutamyl-glutamic acid | L |
| 431.1328 | 28.68 | C517 | 2-(2.4.5-Trimethoxyphenyl)-5.6.7.8-tetramethoxy-4H-1-benzopyran-4-one | P |
| 490.1705 | 28.69 | C518 | Demethylalangiside | L |
| 775.2828 | 28.75 | C519 | Unknown | P |
| 649.2125 | 28.79 | C520 | Unknown | L |
| 693.2037 | 28.79 | C521 | Unknown | L |
| 284.1132 | 28.84 | C522 | Unknown | L |
| 404.1698 | 28.84 | C523 | Cytomycin | L |
| 516.1217 | 28.85 | C524 | Unknown | P |
| 515.1192 | 28.86 | C525 | Dexamethasone sodium phosphate | P |
| 491.1180 | 28.87 | C526 | Aurantio-obtusin beta-D-glucoside | P |
| 771.2358 | 28.96 | C527 | Unknown | L |
| 557.2378 | 28.97 | C528 | Unknown | P |
| 386.1168 | 28.97 | C529 | Dimethomorph | L, P |
| 224.0645 | 28.98 | C530 | Unknown | L, P |
| 570.1904 | 28.98 | C531 | Unknown | L, P |
| 571.1929 | 28.98 | C532 | Unknown | L, P |
| 569.1875 | 28.98 | C533 | Decuroside III | L, P |
| 223.0615 | 28.98 | C534 | Sinapate | L, P |
| 137.0605 | 28.99 | C535 | 3-Methoxybenzyl alcohol | L, P |
| 385.1138 | 28.99 | C536 | 1-O-Sinapoyl-beta-D-glucose | L, P |
| 431.1177 | 28.99 | C537 | Unknown | L, P |
| 559.1561 | 29.02 | C538 | Unknown | L, P |
| 779.2770 | 29.04 | C539 | Unknown | L |
| 559.1521 | 29.06 | C540 | Unknown | P |
| 179.0687 | 29.13 | C541 | Coniferyl alcohol | L |
| 560.1488 | 29.16 | C542 | Unknown | P |
| 337.0924 | 29.16 | C543 | 1-Caffeoyl-4-deoxyquinic acid | P |
| 559.1458 | 29.16 | C544 | Unknown | P |
| 711.1939 | 29.21 | C545 | Bougainvillein-r-I | P |
| 577.1913 | 29.50 | C546 | Podorhizol beta-D-glucoside | L, P |
| 607.2017 | 29.53 | C547 | Unknown | P |
| 475.1582 | 29.80 | C548 | Calcium pantothenate | P |
| 566.1575 | 29.83 | C549 | Unknown | P |
| 565.1554 | 29.83 | C550 | Unknown | P |
| 579.1707 | 30.03 | C551 | Mulberrofuran C | P |
| 491.2117 | 30.19 | C552 | Unknown | P |
| 669.2400 | 30.61 | C553 | Unknown | P |
| 504.1542 | 30.75 | C554 | Unknown | L, P |
| 447.2221 | 30.89 | C555 | Atractyloside A | L, P |
| 622.0979 | 31.16 | C556 | Unknown | L, P |
| 559.1575 | 31.18 | C557 | Unknown | L, P |
| 362.1329 | 31.29 | C558 | Unknown | L, P |
| 363.1344 | 31.29 | C559 | Pyridaben | L, P |
| 526.1897 | 31.29 | C560 | Unknown | L, P |
| 523.0769 | 31.30 | C561 | Unknown | L, P |
| 523.8708 | 31.30 | C562 | Unknown | L, P |
| 525.1880 | 31.30 | C563 | Unknown | L, P |
| 524.1846 | 31.30 | C564 | Unknown | L, P |
| 292.0903 | 31.30 | C565 | Triadimefon | L, P |
| 260.1005 | 31.30 | C566 | Imazapyr | L, P |
| 523.1813 | 31.31 | C567 | Unknown | L, P |
| 524.0347 | 31.31 | C568 | Unknown | L, P |
| 259.0975 | 31.33 | C569 | 8-Deoxylactucin | L, P |
| 361.1291 | 31.33 | C570 | Nagilactone C | L, P |
| 291.0877 | 31.34 | C571 | Unknown | L, P |
| 559.1572 | 31.40 | C572 | Unknown | L, P |
| 622.0982 | 31.44 | C573 | Unknown | L, P |
| 633.2181 | 31.45 | C574 | Unknown | L |
| 863.2632 | 31.64 | C575 | Unknown | P |
| 603.2189 | 31.66 | C576 | Unknown | L |
| 701.1388 | 31.66 | C577 | Unknown | L |
| 602.2167 | 31.66 | C578 | Unknown | L, P |
| 197.0826 | 31.66 | C579 | cis-2.3-Dihydroxy-2.3-dihydro-p-cumate | L |
| 601.2139 | 31.67 | C580 | Unknown | L, P |
| 654.1244 | 31.68 | C581 | Unknown | L |
| 554.1952 | 32.05 | C582 | Unknown | P |
| 391.1396 | 32.06 | C583 | Eupatoroxin | P |
| 321.0975 | 32.06 | C584 | Unknown | P |
| 589.1685 | 32.06 | C585 | Unknown | P |
| 289.1075 | 32.06 | C586 | N-Succinyl-LL-2.6-diaminoheptanedioate | P |
| 392.1422 | 32.07 | C587 | Unknown | P |
| 553.1927 | 32.07 | C588 | Osthenol-7-O-beta-D-gentiobioside | L, P |
| 583.2174 | 32.12 | C589 | Magnesium protoporphyrin | L, P |
| 555.2031 | 32.19 | C590 | Unknown | L, P |
| 445.1485 | 32.44 | C591 | Unknown | P |
| 447.2221 | 32.45 | C592 | Atractyloside A | L, P |
| 446.1805 | 32.66 | C593 | Unknown | L |
| 591.2072 | 32.74 | C594 | Unknown | L, P |
| 505.1701 | 32.77 | C595 | Unknown | P |
| 375.1631 | 32.92 | C596 | Unknown | L |
| 374.1605 | 32.92 | C597 | 6-O-Methyl-N-deacetylisoipecoside aglycon | L |
| 256.0972 | 32.92 | C598 | Tolmetin | L |
| 213.0766 | 33.17 | C599 | Oxaburimamide | L |
| 675.2295 | 33.24 | C600 | Icariin | L, P |
| 577.2314 | 33.51 | C601 | Unknown | P |
| 681.2185 | 33.62 | C602 | Unknown | P |
| 341.1383 | 33.68 | C603 | Phaseollidin hydrate | P |
| 271.0606 | 34.14 | C604 | Naringenin | P |
| 557.2233 | 34.92 | C605 | Unknown | L, P |
| 507.1859 | 34.96 | C606 | Gibberellin 2-O-beta-D-glucoside | P |
| 496.1965 | 35.70 | C607 | Mafoprazine mesylate | L |
| 376.1390 | 35.76 | C608 | Unknown | L |
| 617.2451 | 35.80 | C609 | Unknown | L, P |
| 471.1757 | 35.94 | C610 | 5-Formiminotetrahydrofolate | L |
| 547.1847 | 36.03 | C611 | Flavanone 7-O-[alpha-L-rhamnosyl-(1->2)-beta-D-glucoside] | L |
| 645.2182 | 36.27 | C612 | Unknown | L, P |
| 285.0397 | 36.57 | C613 | Luteolin | L, P |
| 496.1963 | 38.27 | C614 | Mafoprazine mesylate | L |
| 300.0587 | 38.74 | C615 | 1-Guanidino-1-deoxy-scyllo-inositol 4-phosphate | P |
| 299.0556 | 38.75 | C616 | Chrysoeriol | P |
| 284.0319 | 38.75 | C617 | Riccionidin A | P |
| 629.2236 | 39.54 | C618 | 7(1)-Hydroxychlorophyllide a | L, P |
| 955.3079 | 39.83 | C619 | Unknown | L |
| 911.3098 | 39.83 | C620 | Unknown | L, P |
| 910.3082 | 39.84 | C621 | Unknown | L, P |
| 945.2782 | 39.84 | C622 | Unknown | L |
| 909.3056 | 39.84 | C623 | Unknown | L, P |
| 223.0281 | 40.26 | C624 | Sideretin | P |
| 510.2120 | 40.56 | C625 | omega-Carboxy-N-acetyl-LTE4 | L |
| 266.1025 | 40.75 | C626 | 7H-Dibenzo[c.g]carbazole | L |
| 386.1594 | 40.76 | C627 | Nemonapride | L |
| 465.1541 | 41.22 | C628 | Unknown | L, P |
| 627.2088 | 41.22 | C629 | Chlorophyllide b | L, P |
| 361.1461 | 42.01 | C630 | Unknown | L |
| 579.1183 | 42.01 | C631 | Unknown | L |
| 482.2072 | 42.01 | C632 | Unknown | L |
| 480.2021 | 42.02 | C633 | Unknown | L |
| 360.1445 | 42.02 | C634 | 3-Acetylnerbowdine | L |
| 481.2047 | 42.03 | C635 | Unknown | L |
| 401.1989 | 43.48 | C636 | Cortisone acetate | P |
| 512.2277 | 43.48 | C637 | N-Acetylpuromycin | L |
| 541.6854 | 43.94 | C638 | Unknown | L |
| 428.1696 | 44.00 | C639 | Unknown | L |
| 461.2069 | 44.32 | C640 | Patrinoside | L |
| 416.1819 | 44.64 | C641 | Casimiroedine | P |
| 415.1783 | 44.67 | C642 | Erioflorin methacrylate | P |
| 925.3932 | 44.73 | C643 | Unknown | L |
| 356.1495 | 45.62 | C644 | Deacetylcolchicine | L |
| 335.2216 | 45.67 | C645 | Prostaglandin B1 | P |
| 358.1287 | 46.26 | C646 | Clethodim | L |
| 314.1392 | 46.26 | C647 | 3'-Hydroxy-N-methyl-(S)-coclaurine | L |
| 478.1862 | 46.27 | C648 | Unknown | L |
| 540.2226 | 46.73 | C649 | Unknown | L |
| 487.3411 | 47.64 | C650 | Asiatic acid | L, P |
| 342.1344 | 47.75 | C651 | Unknown | L |
| 562.1158 | 47.75 | C652 | Unknown | L |
| 116.9279 | 47.75 | C653 | Unknown | L |
| 462.1916 | 47.75 | C654 | Unknown | L |
| 464.2063 | 48.25 | C655 | Unknown | L |
| 675.3596 | 48.62 | C656 | Unknown | L |
| 721.3656 | 48.62 | C657 | Unknown | L |
